# Metalloprotease-disintegrin ADAM12 actively promotes the stem cell-like phenotype in claudin-low breast cancer

**DOI:** 10.1186/s12943-017-0599-6

**Published:** 2017-02-01

**Authors:** Sara Duhachek-Muggy, Yue Qi, Randi Wise, Linda Alyahya, Hui Li, Jacob Hodge, Anna Zolkiewska

**Affiliations:** 1 Department of Biochemistry and Molecular Biophysics, Kansas State University, 141 Chalmers Hall, Manhattan, KS 66506 USA; 2 Current address: Department of Radiation Oncology, David Geffen School of Medicine, UCLA, Los Angeles, CA USA; 3 Current address: Department of Diabetes Complications and Metabolism, Beckman Research Institute, City of Hope, Duarte, CA USA; 4 Current address: Department of Molecular Biology, University of Texas Southwestern Medical Center, Dallas, TX USA

**Keywords:** Breast cancer, Cancer stem cells, Epidermal growth factor receptor, Epithelial-to-mesenchymal transition, ADAM metalloprotease

## Abstract

**Background:**

ADAM12 is upregulated in human breast cancers and is a predictor of chemoresistance in estrogen receptor-negative tumors. ADAM12 is induced during epithelial-to-mesenchymal transition, a feature associated with claudin-low breast tumors, which are enriched in cancer stem cell (CSC) markers. It is currently unknown whether ADAM12 plays an active role in promoting the CSC phenotype in breast cancer cells.

**Methods:**

ADAM12 expression was downregulated in representative claudin-low breast cancer cell lines, SUM159PT and Hs578T, using siRNA transfection or inducible shRNA expression. Cell characteristics commonly associated with the CSC phenotype in vitro (cell migration, invasion, anoikis resistance, mammosphere formation, ALDH activity, and expression of the CD44 and CD24 cell surface markers) and in vivo (tumor formation in mice using limiting dilution transplantation assays) were evaluated. RNA sequencing was performed to identify global gene expression changes after ADAM12 knockdown.

**Results:**

We found that sorted SUM159PT cell populations with high ADAM12 levels had elevated expression of CSC markers and an increased ability to form mammospheres. ADAM12 knockdown reduced cell migration and invasion, decreased anoikis resistance, and compromised mammosphere formation. ADAM12 knockdown also diminished ALDEFLUOR^+^ and CD44^hi^/CD24^-/lo^ CSC-enriched populations in vitro and reduced tumorigenesis in mice in vivo. RNA sequencing identified a significant overlap between ADAM12- and Epidermal Growth Factor Receptor (EGFR)-regulated genes. Consequently, ADAM12 knockdown lowered the basal activation level of EGFR, and this effect was abolished by batimastat, a metalloproteinase inhibitor. Furthermore, incubation of cells with exogenously added EGF prevented the downregulation of CD44^hi^/CD24^-/lo^ cell population by ADAM12 knockdown.

**Conclusions:**

These results indicate that ADAM12 actively supports the CSC phenotype in claudin-low breast cancer cells via modulation of the EGFR pathway.

**Electronic supplementary material:**

The online version of this article (doi:10.1186/s12943-017-0599-6) contains supplementary material, which is available to authorized users.

## Background

Members of the ADAM family of cell surface metalloproteases catalyze cell context-dependent cleavage of transmembrane receptors, growth factor precursors, or adhesion molecules [1, 2]. ADAM substrates include many cancer-related proteins, such as Notch receptors and their ligands [3], epidermal growth factor receptor (EGFR) ligands [4, 5], interleukin-6 receptor (IL-6R) [6], tumor necrosis factor (TNF) and its receptors [7], E-cadherin [8], and CD44 [9]. Because ADAMs are often aberrantly expressed or misregulated in human cancers, they may contribute to tumor progression, metastasis, or therapy resistance [10–12].

Among twelve catalytically active human ADAMs [2], ADAM12 possesses unique characteristics that make it an attractive candidate for future use as a target or a biomarker in breast cancer. ADAM12 expression is strongly elevated in most human breast cancers compared to normal mammary epithelium [13, 14]. *ADAM12* mRNA is alternatively spliced, and high levels of *ADAM12* transcript variant 1 (encoding the transmembrane protein isoform ADAM12-L) are associated with poor prognosis and decreased metastasis-free survival times in estrogen receptor (ER)-negative, progesterone receptor (PR)-negative, and human epidermal growth factor receptor 2 (HER2)-negative (triple-negative) early stage breast cancers without systemic treatment, but not in HER2-positive or ER-positive tumors [15, 16]. ADAM12-L expression is induced during epithelial-to-mesenchymal transition (EMT) in mammary epithelial cells [17] and appears to be upregulated in the claudin-low intrinsic subtype of breast cancer [18], which harbors molecular signatures of EMT. Claudin-low tumors represent ~5-10% of all breast cancers, are often triple-negative and poorly differentiated, and have elevated activities of EGFR, proto-oncogene tyrosine kinase Src, transforming growth factor β (TGFβ), and signal transducer and activator of transcription 3 (STAT3) pathways [19–21]. Importantly, the gene expression signatures of claudin-low tumors show a significant similarity to the signature of CD44^hi^/CD24^-/lo^ mammosphere-forming cells [20, 22], suggesting an enrichment in cancer stem cell (CSC)-like or tumor-initiating cell features. Breast CSCs are thought to be largely responsible for tumor maintenance, treatment resistance, and disease recurrence [23–25]. Our previous analysis of two clinical datasets showed that elevated expression of *ADAM12* mRNA is predictive of resistance to neoadjuvant chemotherapy in ER-negative breast cancer, independent of age, tumor size, grade, and the lymph node status [18]. These observations raise a possibility that ADAM12 may serve as a marker or a therapeutic target in CSCs in ER-negative or triple-negative breast cancer (TNBC).

The goal of the current study was to assess a possible contribution of ADAM12 to the CSC phenotype of claudin-low TNBC cells. By comparing the properties of sorted cell populations with high versus medium expression of ADAM12, and by analyzing the effect of ADAM12 knockdown on cell migration, invasion, anoikis resistance, mammosphere formation, known CSC markers, tumor formation after xenotransplantation in mice in vivo, and global gene expression, we have determined that ADAM12 actively supports the CSC phenotype of claudin-low TNBC cells. This function of ADAM12 appears to be mediated by sustained, ligand-dependent activation of EGFR. Thus, we have identified ADAM12 as an important modifier of the EGFR pathway in claudin-low TNBC and a potential target in CSC-directed therapies.

## Methods

### Reagents and antibodies

SMARTpool ADAM12 siRNA (M-005118-01, target sequences 5’-GCAAAGAACTGATCATAAA-3’, 5’-GATGAGAGATGCTAAATGT-3’, 5’-GCAGCAAGGAGGCCGGATT-3’, and 5’-GTCAGGATGTGGACGGCTA-3’), ADAM12 siRNA#1 (D-005118-01, target sequence 5’-GCAAAGAACTGATCATAAA-3’), ADAM12 siRNA#2 (D-005118-02, target sequence 5’-GATGAGAGATGCTAAATGT-3’), and DharmaFECT1 transfection reagent were from GE Dharmacon. These siRNAs targeted transcript variant 1 (NCBI Ref. Seq. NM_003474) and transcript variant 2 (NCBI Ref. Seq. NM_021641) of *ADAM12*, encoding ADAM12-L and ADAM12-S protein isoforms, respectively. As a negative control, siGENOME non-targeting siRNA pool (D-001206-13) or ON-TARGETplus non-targeting siRNA pool (D-001810-10, GE Dharmacon) was used. RT^2^ Profiler arrays were from Qiagen, erlotinib was from Cell Signaling Technology, batimastat was from EMD Millipore, bFGF, B27, and human recombinant EGF were from Life Technologies. Antibodies used for flow cytometry were: PE-conjugated anti-CD24 (clone ML5) and IgG2aκ isotype control (clone G155-178, both from BD Biosciences), APC-conjugated anti-CD44 (clone IM7) and IgG2bκ isotype control (clone eB149/10H5, both from Affymetrix eBioscience), anti-ADAM12 (clone 632525) and IgG1 isotype control (clone 11711, both from R&D Systems). APC- or AlexaFluor488-conjugated secondary anti-mouse IgG1 antibodies were from Jackson ImmunoResearch. For Western blotting, the following antibodies were used: rabbit monoclonal anti-pY1068 EGFR (clone D7A5) and anti-total EGFR (clone D38B1), both from Cell Signaling Technology, and rabbit polyclonal anti-ADAM12 antibody (Ab#3394) raised against the cytoplasmic tail of human ADAM12 [16].

### Cell culture

SUM149PT, SUM159PT, and SUM1315MO2 cell lines were purchased from Asterand, BT549, Hs578T, and MCF-7 cells were from ATCC, and HEK293T cells were from Thermo Scientific. SUM102PT and SUM225CWN cells were gifts from Dr. Fariba Behbod (University of Kansas Medical Center). All cell lines were authenticated by the original suppliers using the short tandem repeat (STR) analysis and have been passaged for fewer than 6 months after culture initiation from an early passage number. Tumor source and pathological features of tumors used to derive breast cancer cell lines used in this study are summarized in Additional file 1: Table S1 [19, 26–29]. SUM149PT, SUM159PT, and SUM225CWN cells were cultured in Ham’s F-12 medium supplemented with 5% fetal bovine serum (FBS), 10 mM HEPES, 5 μg/ml insulin, and 1 μg/ml hydrocortisone. SUM1315MO2 cells were cultured in Ham’s F-12 medium supplemented with 5% FBS, 10 mM HEPES, 10 ng/ml EGF, and 5 μg/ml insulin. SUM102PT cells were maintained in Ham’s F-12 medium containing 5% FBS, 1 μg/ml hydrocortisone, and 5 μg/ml insulin. BT549 cells were cultured in RMPI1640 medium supplemented with 10% FBS, 1 mM pyruvate, and 0.8 μg/ml insulin. Hs578T and MCF-7 cells were cultured in DMEM medium containing 10% FBS and 10 μg/ml insulin. HEK293T cells were cultured in DMEM medium containing 10% FBS, 6 mM glutamine, and 1 mM pyruvate. Cells were maintained at 37 °C under humidified atmosphere containing 5% CO_2_.

### Virus production and generation of stable cell lines

shRNA targeting human ADAM12 and control shRNA inserts were excised from GIPZ ADAM12 shRNA lentiviral vector (V2LHS_11814; the mature antisense sequence: 5’-TTGACATTGACGATTCAGG-3’) or non-silencing control shRNA construct (RHS4346; GE Dharmacon), respectively, and were cloned into pInducer10 vector (Addgene, plasmid 44011) at the XhoI and MluI sites. The ADAM12 shRNA construct targeted both transcript variant 1 and 2 of *ADAM12*. Lentiviruses were produced by transfecting HEK293T cells with pInducer10-shADAM12/shControl, pMD2.G, and psPAX2 (Addgene, plasmids 12259 and 12260, respectively) using Mirus TransIT transfection reagent (Mirus). Conditioned media containing viral particles were harvested 48 h after transfection, supplemented with 5 μg/ml polybrene (Sigma), and added onto SUM159PT cells at ~20% confluence. Selection of stably transduced cells started 48 h after infection using 2 μg/ml puromycin and continued for 10 days.

### Flow cytometry

*ALDEFLUOR assay*: SUM159PT cells and Hs578T cells were transfected with SMARTpool ADAM12 siRNAs or non-targeting siRNAs. Three days after transfection, 10^6^ cells were suspended in 1 ml ALDEFLUOR assay buffer and 5 μl ALDEFLUOR reagent (STEMCELL) was added. Then, 0.5 ml cells were immediately transferred to a new tube containing 10 μl ALDH inhibitor diethylamino-benzaldehyde (DEAB, negative control). Both DEAB-treated and untreated cells were incubated at 37 °C for 45 min, centrifuged, resuspended, and analyzed by flow cytometry. *CD24 and CD44 staining*: Three days after transfection, cells were stained with PE-conjugated anti-CD24 and APC-conjugated anti-CD44 antibodies, or their respective isotype antibody controls, and analyzed by flow cytometry. *ADAM12 staining*: Cells were incubated with anti-ADAM12 antibody or mouse IgG1 isotype control antibody, washed and incubated with APC-conjugated anti-mouse IgG antibody. Cells were analyzed with a BD FACSCalibur cytometer or a LSR Fortessa X20 instrument. All data were analyzed with FCS Express 4 (DeNovo Software). For cell sorting, single cell suspensions stained with anti-ADAM12 antibody and with AlexaFluor488-conjugated anti-mouse IgG antibody were sorted on Bio-Rad S3 cell sorter into ADAM12^hi^ population (~1.5% of cells with the highest expression of ADAM12) and ADAM12^med^ population (medium expression of ADAM12).

### Transwell migration and matrigel invasion assays

SUM159PT shADAM12 and shControl cells were treated with or without 1 μg/ml doxycycline for 4 days. Transwell migration assay was then performed, as described [30]. Migrated cells underneath the inserts were analyzed by light microscopy using 10x magnification and photographed. The numbers of migrated cells were counted in five random fields for each insert. For invasion assays, growth factor-reduced Matrigel (Corning, 200 μl at 6.3 mg/ml) was layered into wells of a 24-well plate and allowed to solidify at 37 °C. Cells were pre-treated for 4 days with or without 1 μg/ml doxycycline, detached, and diluted to 2.5x10^4^ cells/ml. Cells (10 μl) were mixed with 190 μl diluted Matrigel, placed onto the Matrigel base layer, and incubated at 37 °C. After the cell layer solidified, 500 μl of complete growth medium supplemented with 2 μg/ml doxycycline was placed on top and incubated at 37 °C for 12 days. Cells were visualized on a Nikon inverted microscope at 4x magnification and photographed.

### Sphere formation

Cells were treated without or with 1 μg/ml doxycycline for 4 days, detached with 0.25% trypsin/5 mM EDTA in DPBS, and diluted to 1x10^6^ cells/ml. Single cell suspension was passaged through a cell strainer and then diluted to 5x10^3^ cells/ml with mammosphere medium containing MEMB, 20 ng/ml hEGF, 20 ng/ml bFGF, 4 μg/ml heparin, 1xB27, and 1x Penicillin/Streptomycin. Cells were mixed with 3% methylcellulose (R&D Systems) at a 2:1 ratio and seeded into wells of 24-well ultra-low attachment plates (Corning) at 1x10^3^ cells/well. After 7 days, six randomly selected areas from each well were analyzed by inverted light microscopy using a 4x objective and photographed. Numbers of spheres in each image were quantified using ImageJ.

### Cell growth and cell death assays

ADAM12^hi^ and ADAM12^med^ sorted cells were seeded into 96-well plates (100 cells/well) and incubated for 5 days in growth medium. Cell growth was measured using CellTiter-Glo Luminescent Cell Viability Assay (Promega); at least 5 wells per cell type were measured for each time point. Growth rate and doubling times were calculated using GraphPad Prism.

For cell death assays, 1x10^3^ cells treated without or with 1 μg/ml doxycycline for 4 days were suspended in mammosphere medium containing 1% methylcellulose and seeded into wells of a 24-well ultra-low attachment plate. Cells were pelleted at 0, 12, 24, and 48 h post-plating, washed twice, lysed at 4 °C for 15 min, and frozen at -20 °C. Cell Death ELISA kit (Roche) was used to detect fragmented mono- and oligo-nucleosomes in lysates preserved at different time points.

### Immunoblotting

Immunoblotting was performed as described [16, 18], with some modifications. Lysis buffer was supplemented with phosphatase inhibitors (50 mM NaF, 2 mM Na_3_VO_4_, and 10 mM Na_4_P_2_O_7_). Total cell lysates were directly analyzed by Western blotting or incubated with concanavalin A agarose (Sigma; 50 μl resin per 1 ml cell lysate) for 2 h at 4 °C to enrich for glycoproteins. The resin was washed three times and glycoproteins were eluted with 3X SDS gel loading buffer. Nitrocellulose membranes were incubated with primary monoclonal antibodies and HRP-conjugated secondary antibodies, followed by signal detection using SuperSignal West Pico or West Femto chemiluminescence detection kit (Pierce) and Azure c500 digital imaging system. Band intensities were quantified using the ImageJ analysis software.

### Quantitative real-time PCR

Total RNA was extracted using the Qiagen RNeasy kit and was subjected to on-column digestion with deoxyribonuclease I. One microgram of total RNA was reverse-transcribed using the SuperScript III First Strand Synthesis system (Invitrogen) or the qScript Flex cDNA kit (Quanta BioSciences) and oligo(dT) primers. Quantitative real time PCR (qRT-PCR) was performed on a BioRad CFX96 instrument. At the conclusion of each run, a melt curve analysis was performed to ensure that a single product had been synthesized. The relative expression of each gene, normalized to actin, was calculated using the 2^-ΔΔCt^ method. Primer sequences are provided in Additional file 1: Table S7.

### Tumor formation in vivo

All animal experiments were performed in accordance with protocols approved by the Institutional Animal Care and Use Committee at Kansas State University. Non-obese diabetic severe combined immunodeficient (NOD-SCID) female mice were obtained from Charles River Laboratories. One week prior to injection, mice were randomized to either control or doxycycline diet (2 g/kg, Bio-Serv) and they remained on this diet throughout the experiment. shControl and shADAM12 SUM159PT cells were pre-treated for 4 days with or without 1 μg/ml doxycycline. Cells were detached and serially diluted in 1% BSA/DPBS; each dilution was combined with an equal volume of ice-cold growth factor-reduced Matrigel. Cells (100 μl) were injected subcutaneously in the 4th inguinal mammary glands of 8-week old female NOD-SCID mice under isoflurane sedation. Mice were monitored for tumor development twice per week using a caliper. Tumor volumes were determined using the formula: volume = 1/2 × length × width^2^.

### RNA sequencing

Total RNA was isolated from shControl and shADAM12 SUM159PT cells ± doxycycline (n = 3 for each condition) using the Qiagen RNeasy purification kit and on-column DNase I digestion according to the manufacturer’s protocols. The RNA was sent to the Kansas State University Integrated Genomics Facility for quality assessment using the Agilent 2100 Bioanalyzer and library preparation. An NGS library was generated for each RNA sample using the Illumina TruSeq Library Prep kit. The libraries were analyzed at the University of Kansas Medical Center Genome Sequencing Facility on an Illumina HiSeq 2500 with 100 bp single reads. All sequence analysis was performed using the CLC Genomics Workbench version 8.0 (CLC Bio). The reads were trimmed based on the quality score with a limit of 0.05 and ambiguous nucleotides were trimmed from the ends with 2 set as the maximum number of ambiguities allowed. Additionally, sequences shorter than 15 nt or longer than 1000 nt were eliminated. Mapping to the human genome (hg19) was performed using the RNA-Seq Analysis tool and the following settings: map to gene regions only, mismatch cost = 2, insertion and deletion costs = 2, length and similarity fractions = 0.8, and maximum number of hits for a read = 1. The expression value was calculated as Reads Per Kilobase of transcript per Million mapped reads (RPKM). The RPKM values were statistically analyzed using the Empirical analysis of Digital Gene Expression tool (EDGE) and FDR corrected *P*-values were calculated. Genes that were significantly changed by ADAM12 knockdown were further evaluated by QIAGEN’s Ingenuity® Pathway Analysis (IPA®) using the Canonical Pathways and Upstream Regulator features. RNA sequencing data have been deposited to the NCBI Sequence Read Archive (SRA) and are available at the accession number SRP077683.

### Data mining

Expression levels of *ADAM12* (transcript variant 1, NM_003474) in 295 breast tumors from the NKI dataset were retrieved from the Computational Cancer Biology website at The Netherlands Cancer Institute (http://ccb.nki.nl/data/) as ratios of fluorescence intensities to the intensity of a reference pool [31]. Tumors were assigned to individual subtypes of breast cancer according to ref. [32]. Expression data for *ADAM12* in 508 breast invasive carcinomas from The Cancer Genome Atlas (Nature 2012 dataset) [33] were accessed via the cBioPortal for Cancer Genomics (http://www.cbioportal.org/public-portal/) [34, 35]. Since cBioPortal contains only gene-level data and it does not contain probe-level data, *ADAM12* expression values obtained through cBioPortal represent merged data for different *ADAM12* splice variants. *P*-values for a *t*-test of *ADAM12* expression in each TNBC subtype versus all other TNBC subtypes were retrieved from ref. [36]. The list of genes whose expression was most highly correlated with the expression of the *ADAM12* gene was acquired from the TCGA (Nature 2012 dataset) via the cBioPortal using the mRNA co-expression feature. Overlaps between *ADAM12*-correlated genes and gene sets from the MSigDB collection were computed using the GSEA website (http://software.broadinstitute.org/gsea/msigdb/annotate.jsp).

### Calculation of the ADAM12, CSC, and EGFR gene expression signature scores

Genes (*n* = 45) significantly changed after ADAM12 knockdown in SUM159PT cells (Fig. 5b and Additional file 1: Table S5) were used to construct an *ADAM12* gene expression signature. Genes (355 different genes corresponding to 493 individual transcripts) significantly upregulated or downregulated in CD44^hi^/CD24^-/lo^ subpopulations of primary breast cancer cells and in cancer mammospheres [22] were used to calculate the CSC signature scores. Expression values for these CSC-related genes in 51 different breast cancer cell lines were retrieved from Gene Expression Omnibus (GEO, http://www.ncbi.nlm.nih.gov/geo/), using accession number GSE69017. The top 250 genes whose expression was most significantly changed upon stable expression of EGFR in MCF7 cells [37] were used to calculate EGFR-responsive gene signature scores. Expression values for these EGFR-regulated genes were retrieved from GEO, using accession number GSE3542. The expression values for ADAM12-, CSC-, and EGFR-related genes in breast invasive carcinomas were extracted from TCGA (Cell 2015 dataset) [38] via the cBioPortal (http://www.cbioportal.org/public-portal/). The signature scores were then calculated for all tumors for which gene expression values were available (a total of 421 tumors) as:

$$ s={\displaystyle \sum_i{w}_i{x}_i}/{\displaystyle \sum_i\left|{w}_i\right|} $$

where *w* is the weight +1 or –1, depending on whether the gene was upregulated or downregulated in the signature, and *x* is the normalized gene expression level.

### Survival analysis

The effect of ADAM12 expression on relapse-free survival rates of breast cancer patients was assessed using the Kaplan-Meier Plotter (http://kmplot.com/analysis/). This online tool uses manually curated database containing gene expression data and relapse free and overall survival information downloaded from GEO, the European Genome-Phenome Archive (EGA), and TCGA [39]. The parameters were set as follows: Affymetrix probeset: 202952_s_at (as this probeset is specific for *ADAM12* transcript variant 1, encoding ADAM12-L); Auto select best cutoff: On; Use array quality control: Remove redundant samples, Exclude outlier arrays; Check proportional hazards assumption: On; Restrict analysis to subtypes: All, or ER-negative (derive ER status from gene expression data: On), PR-negative, HER2-negative, or Mesenchymal stem-like; Restrict analysis to selected cohorts: none; Database release: 2017; Datasets: all.

## Results

### ADAM12 expression is upregulated in claudin-low breast tumors and in cell populations enriched for breast cancer stem cell-like cells

By analyzing gene expression profiles of 337 breast tumors from the University of North Carolina database, we previously noted that *ADAM12* mRNA levels (transcript variant 1 encoding ADAM12-L protein isoform, but not transcript variant 2 encoding ADAM12-S) were significantly elevated in the claudin-low subtype of breast cancer [18]. Here, we extended our observations to two other tumor gene expression repositories: the Netherlands Cancer Institute database (NKI, 295 breast cancers) [31] and The Cancer Genome Atlas database (TCGA, 508 breast cancers) [33]. In the NKI database, *ADAM12* mRNA levels (transcript variant 1) were more frequently upregulated in claudin-low (CL) tumors compared to basal (B), luminal A/B (LumA/B), HER2-enriched (HER2+), or normal-like (NL) cancers (Additional file 2: Figure S1a). Similarly, *ADAM12* mRNA expression levels in the TCGA database (retrieved as merged values for different splice variants) were significantly higher in claudin-low tumors than in other tumor subtypes (Additional file 2: Figure S1b). Furthermore, among different subtypes of TNBCs [36], *ADAM12* mRNA levels (transcript variant 1) were previously found significantly elevated (*P* = 5.19E-08) in the mesenchymal stem-like (MSL) subtype compared to the mesenchymal (M), basal-like 1 (BL1), basal-like 2 (BL2), immunomodulatory (IM), or luminal androgen receptor (LAR) subtypes (Additional file 1: Table S2). Notably, the MSL subtype shares many features with the claudin-low subtype of breast cancer, including high expression of EMT- and CSC-related genes [40].

Investigation of the top 40 genes which are most closely correlated with *ADAM12* expression in breast invasive carcinomas from the TCGA database (Additional file 1: Table S3) revealed a very significant enrichment (*P* = 7.43E-50) in the EMT hallmark signature from the Gene Set Enrichment Analysis/Molecular Signature Database (GSEA/MSigDB, ref. [41]) (Additional file 1: Table S4). These findings corroborate a recent report, in which *ADAM12* mRNA expression was correlated with EMT markers N-cadherin, vimentin, and TGFβ, but not E-cadherin, in a panel of human breast cancer cell lines and in 79 breast cancer biopsies [17].

**Fig. 1:**
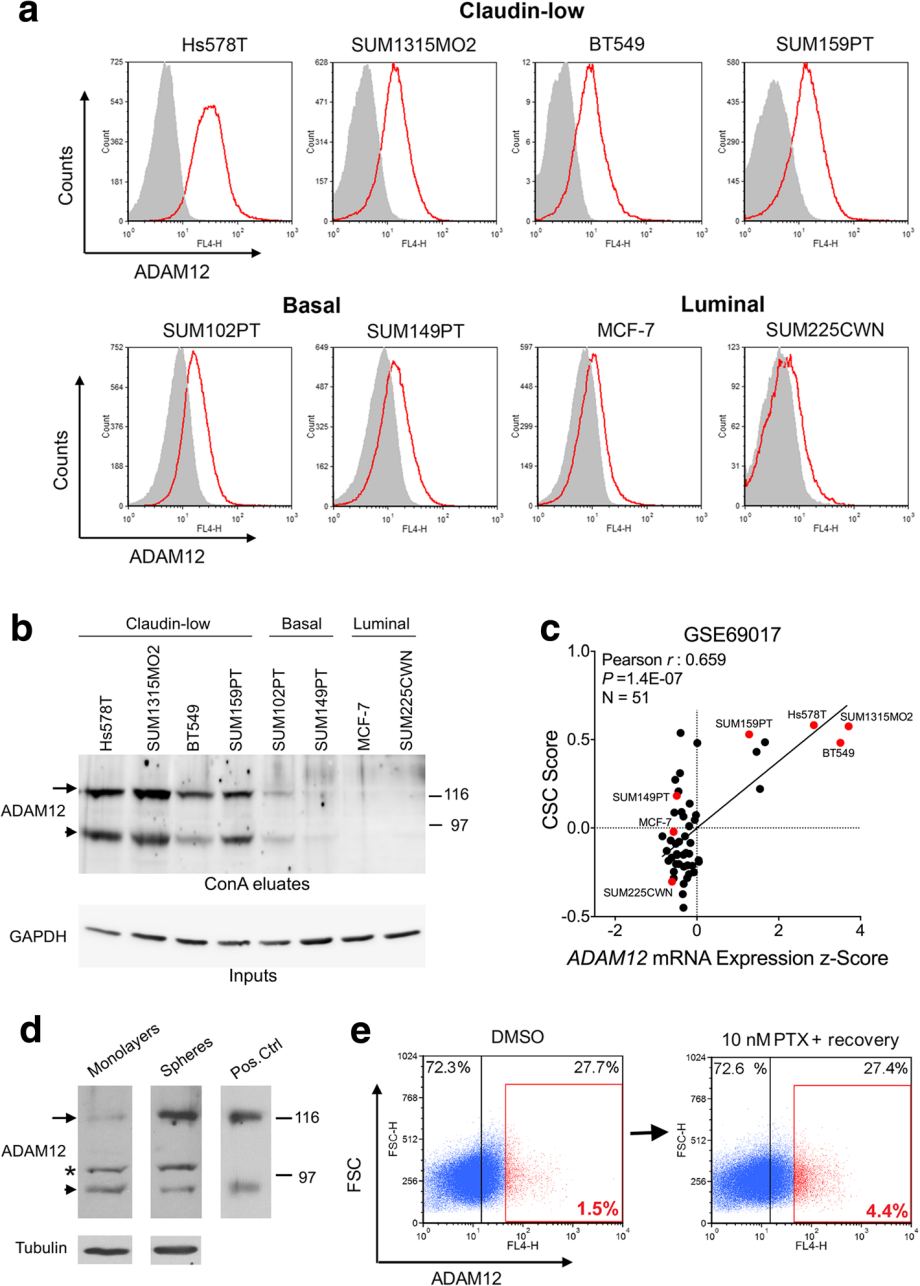
ADAM12 expression is upregulated in claudin-low breast cancer cells and in subpopulations enriched for CSCs. **a**. Cell surface expression of ADAM12 protein in breast cancer cell lines was evaluated by flow cytometry. Red, anti-ADAM12 antibody staining; gray, isotype control antibody staining. **b** Total cellular expression of ADAM12 was analyzed by Western blotting after partial purification of ADAM12 on concanavalin A (conA) agarose, as described [42]. GAPDH in the input fractions shows comparable conA agarose loading for all cells. Arrow, the nascent full length ADAM12; arrowhead, the processed form lacking the N-terminal pro-domain. **c** CSC signature score versus *ADAM12* mRNA expression in 51 breast cancer cell lines. The CSC signature scores were calculated based on ref. [22] and microarray expression data retrieved from GEO:GSE69017, as described in Methods. Cell lines analyzed in panels **a** and **b** are shown in red. **d** ADAM12 protein levels in total lysates of SUM159PT cells grown as attached monolayers or as mammospheres, analyzed by Western blotting. Arrow, the nascent full length ADAM12; arrowhead, the processed form lacking the N-terminal pro-domain; *, a non-specific band. Positive control represents ADAM12 after partial purification on conA agarose. **e** Cell surface expression of ADAM12 in SUM159PT cells treated for 6 days with DMSO (control) or with 10 nM paclitaxel (PTX), and then allowed to recover for 6 days without PTX, was examined by flow cytometry. The population of cells with the highest expression of ADAM12 is shown in red. FSC, forward scatter

Here, we examined ADAM12 protein levels in several cell lines representing different intrinsic subtypes of breast cancer: claudin-low cell lines Hs578T, SUM1315MO2, BT549, and SUM159PT, basal cell lines SUM102PT and SUM149PT, and luminal cell lines MCF-7 and SUM225CWN [19]. Cell surface levels of ADAM12-L were evaluated by flow cytometry after staining of live cells with an antibody specific for the extracellular domain of ADAM12-L (Fig. 1a). Also, total cellular levels of ADAM12-L, after partial purification on concanavalin A agarose, were detected by Western blotting using an antibody specific for the cytoplasmic domain of ADAM12-L (Fig. 1b). The specificity of ADAM12 bands was confirmed after ADAM12 knockdown using an inducible shRNA construct (see below). In accordance with *ADAM12* mRNA expression data [17, 42], ADAM12-L protein (from now on referred to as ADAM12) was most abundantly expressed in claudin-low cell lines.

Since the gene expression signatures of claudin-low tumors and claudin-low cell lines show a significant similarity to the signature of CSCs [20, 22], we examined the relationship between *ADAM12* mRNA expression and the CSC signature scores in a panel of 51 breast cancer cell lines from Neve at al. [26]. The CSC scores were calculated based on the genes whose expression was most significantly changed in CD44^hi^/CD24^-/lo^ populations and in mammosphere-forming cells, as reported by Creighton et al. [22] and described in Methods. As shown in Fig. 1c, there was a significant positive correlation between CSC scores and *ADAM12* mRNA levels (*P* = 1.4E-07).

One of the characteristic features associated with the CSC phenotype is the resistance to anoikis, an apoptotic cell death upon matrix detachment. When SUM159PT cells were grown as mammospheres in ultra-low attachment plates in serum-free medium, ADAM12 was more abundant in mammospheres than in attached cells grown as monolayers (Fig. 1d, see also [18]). Since mammosphere-forming cells are largely anoikis-resistant, this result suggested that ADAM12 expression might be upregulated in breast CSCs.

Another characteristic feature of CSCs is their resistance to chemotherapy. It was shown previously that overexpression of ADAM12 in MCF10A cells, a non-transformed human breast epithelial cell line, increased cell resistance to cisplatin [17]. Here, we asked whether cells with high endogenous expression of ADAM12 would preferentially survive treatment with a chemotherapeutic agent. To this end, non-confluent adherent cultures of SUM159PT cells were treated for 6 days with 10 nM paclitaxel, which induced death in ~90% of cells. The surviving ~10% of cells were subsequently incubated for 6 additional days in the absence of paclitaxel, during which they resumed proliferation (a “recovery phase”). In DMSO-treated cultures, ~1.5% of cells expressed ADAM12 at the level that was equal to or exceeded an arbitrarily defined “high” expression level (ADAM12^hi^), whereas in paclitaxel-treated cultures, the percentage of ADAM12^hi^ cells was increased ~3-fold (Fig. 1e and Additional file 3: Figure S2). This result indicated that cells with high expression of ADAM12 might be more chemoresistant than cells with lower ADAM12 expression, as it appears that they preferentially survived paclitaxel treatment.

Using fluorescence-activated cell sorting, we next isolated two subpopulations of SUM159PT cells: ADAM12^hi^ representing ~1.5% of cells with the highest cell surface expression of ADAM12, and ADAM12^med^ representing cells with medium ADAM12 expression levels (Additional file 4: Figure S3). ADAM12^hi^ cells grew slower than ADAM12^med^ cells under standard 2D conditions in complete growth medium (doubling time 19 ± 0.9 h versus 14 ± 0.2 h, *P* < 0.0001, Additional file 5: Figure S4a). However, ADAM12^hi^ cells formed more primary and secondary mammospheres than ADAM12^med^ cells (Additional file 5: Figure S4b). ADAM12^hi^ cells also expressed higher levels of several CSC markers, mesenchymal markers, and cancer drug resistance genes than ADAM12^med^ cells (Additional file 5: Figure S4c-e, respectively). These results indicated that, in SUM159PT cells, a subpopulation characterized by high endogenous expression levels of ADAM12 was enriched in features commonly attributed to CSCs.

### ADAM12 knockdown reduces stem cell-like features of breast cancer cells in vitro

**Fig. 2:**
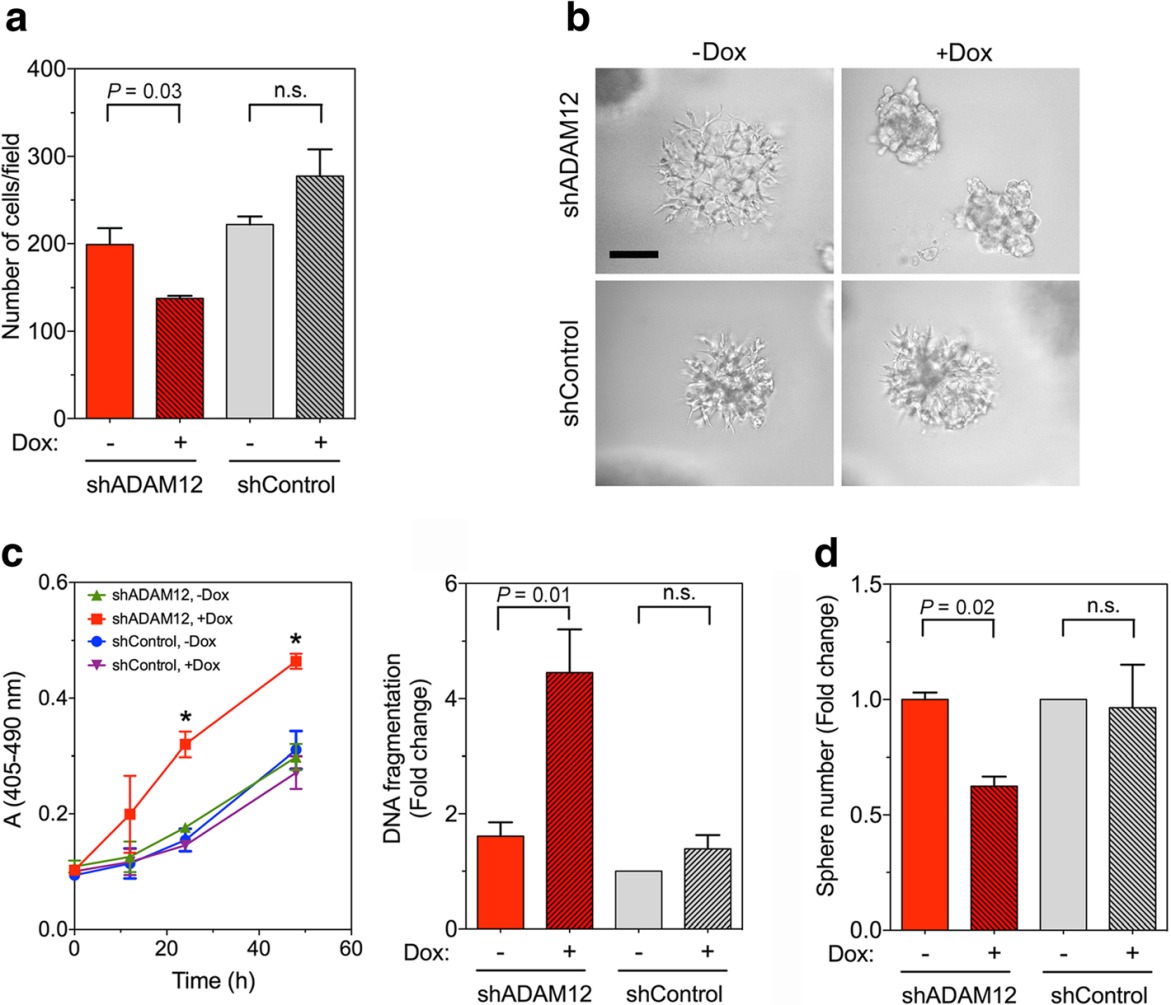
ADAM12 knockdown reduces cell migration, invasion, resistance to anoikis, and sphere formation. SUM159PT cells expressing inducible ADAM12 shRNA (shADAM12) or control shRNA (shControl) were treated with doxycycline (Dox; 1 μg/ml) for 4 days. **a** Cell migration was analyzed using Transwell assays. The data are shown as means ± SEM from 3 determinations. n.s., non-significant, *P* > 0.05. **b** Representative phase-contrast images of SUM159PT_shADAM12 and SUM159PT_shControl cells embedded in 3D Matrigel. Scale bar, 100 μm. **c** Anoikis (apoptotic cell death upon detachment) was assayed using a DNA fragmentation ELISA kit. A time course of cell death was measured in triplicates (raw data, left), and the mean values of DNA fragmentation, ±SEM, after 24 h of detachment obtained in three independent experiments were calculated (right). *, *P* < 0.05. **d** Sphere formation was evaluated after seeding single-cell suspensions in serum-free sphere medium containing 1% methylcellulose into ultra-low attachment plates. After 7 days, spheres with diameters >50 μm were photographed and counted using ImageJ. The results are shown as mean fold change in sphere numbers after doxycycline treatment, normalized to shControl cells without doxycycline, ±SEM, obtained in 3 independent experiments

To examine whether ADAM12 plays an active role in supporting the CSC phenotype, we first examined the effect of ADAM12 knockdown on cell migration, invasion, and mammosphere formation using a doxycycline-inducible shRNA system. ADAM12 shRNA (shADAM12) or control shRNA (shControl) cloned into pInducer10 vector (Additional file 6: Figure S5a) was stably transduced into SUM159PT cells. Treatment with 1 μg/ml doxycycline for 4 days significantly decreased the level of *ADAM12* mRNA (Additional file 6: Figure S5b) and protein (Additional file 6: Figure S5c, d) in SUM159PT_shADAM12 cells, but not in SUM159PT_shControl cells. ADAM12 knockdown did not have a significant effect on cell proliferation or survival rates (results not shown). In the Transwell migration assay, SUM159PT_shADAM12 cells treated with doxycycline were less motile than untreated cells (Fig. 2a). In three-dimensional Matrigel cultures, doxycycline-treated SUM159PT_shADAM12 cells were less invasive than shADAM12 cells incubated without doxycycline (Fig. 2b). Doxycycline treatment did not have a significant effect on the migration or invasion of SUM159PT_shControl cells (Fig. 2a, b). Furthermore, induction of ADAM12 knockdown decreased the ability of SUM159PT cells to survive under detachment conditions. Using an apoptosis assay based on measuring DNA fragmentation by ELISA, we observed an increased amount of cell death in doxycycline-treated detached SUM159PT_shADAM12, but not in SUM159PT_shControl cells (Fig. 2c). Accordingly, the number of mammospheres formed by doxycycline-treated SUM159PT_shADAM12 cells was lower than the number of mammospheres formed by untreated SUM159PT_shADAM12 cells or by SUM159PT_shControl cells (Fig. 2d). Thus, ADAM12 knockdown decreased the migration, invasion, anoikis-resistance, and mammosphere-forming potential of SUM159PT cells.

**Fig. 3:**
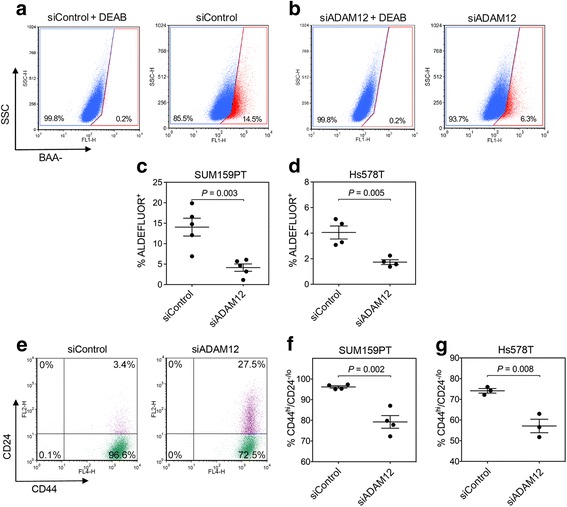
ADAM12 knockdown reduces the ALDEFLUOR+ subpopulation and diminishes the CD44^hi^/CD24^-/lo^ subpopulation of cells. **a**, **b** SUM159PT cells were transfected with a pool of four control siRNAs (siControl, **a**) or a pool of four ADAM12 siRNAs (siADAM12, **b**) and analyzed three days later by flow cytometry. Plots show side cell scatter (SSC) versus BAA^-^ fluorescence (BODIPY-aminoacetate, an intracellular reaction product of aldehyde dehydrogenase). ALDEFLUOR^-^ (blue) and ALDEFLUOR^+^ (red) gates were identified after pre-treatment of cells with DEAB, an ALDH inhibitor. **c**, **d** Percentages of ALDEFLUOR^+^ populations in SUM159PT (**c**) and Hs578T (**d**) cells were determined in at least four independent experiments; shown are means ± SEM. **e** SUM159PT cells transfected with pooled siRNAs were stained with anti-CD24-PE and anti-CD44-APC antibodies and analyzed by flow cytometry. **f**, **g** Percentages of CD44^hi^/CD24^-/lo^ populations in SUM159PT cells (**f**) and Hs578T cells (**g**) were determined in at least three independent experiments; shown are means ± SEM

Breast CSCs are characterized by high levels of aldehyde dehydrogenase (ALDH) activity and have CD44^hi^/ CD24^-/lo^ cell surface marker profile [23, 43]. To evaluate the effect of ADAM12 knockdown on ALDH (measured as ALDEFLUOR^+^ cells), CD44, and CD24 expression, we used flow cytometry-based assays. To minimize possible inaccuracies that might be introduced during compensation protocols (required to correct for spectral overlaps between different fluorochromes and a strong fluorescence signal of red fluorescent protein (RFP), which was co-expressed with shRNAs from the pInducer10 vector), we used siRNA transfection to knockdown ADAM12. First, SUM159PT cells were transfected with a pool of four siRNAs targeting ADAM12, all of which had different ADAM12-targeting sequences than shADAM12. Downregulation of ADAM12 at the cell surface was confirmed by flow cytometry (Additional file 7: Figure S6a). The amount of ALDEFLUOR^+^ cells was decreased ~3-fold in siADAM12-transfected SUM159PT cells compared to siControl-transfected cells (Fig. 3a-c). A similar effect of ADAM12 knockdown was observed in Hs578T cells, in which the number of ALDEFLUOR^+^ cells was diminished ~2-fold upon siADAM12 transfection (Fig. 3d). An efficient ADAM12 knockdown in siADAM12-transfected Hs578T cells was confirmed by flow cytometry (Additional file 7: Figure S6b).

ADAM12 knockdown using pooled siRNAs resulted in a significant reduction of the CSC-containing CD44^hi^/CD24^-/lo^ population and expansion of the non-CSC CD44^hi^/CD24^+^ population in SUM159PT cells (Fig. 3e, f) and Hs578T cells (Fig. 3g). Increased expression of CD24 (and thus an expansion of the non-CSC population) was also observed in shADAM12-expressing SUM159PT cells treated with doxycycline, but not in shControl-expressing SUM159PT cells upon the same treatment (Additional file 8: Figure S7a and b, respectively).

### ADAM12 knockdown decreases tumor initiating potential in mouse xenografts in vivo

**Fig. 4:**
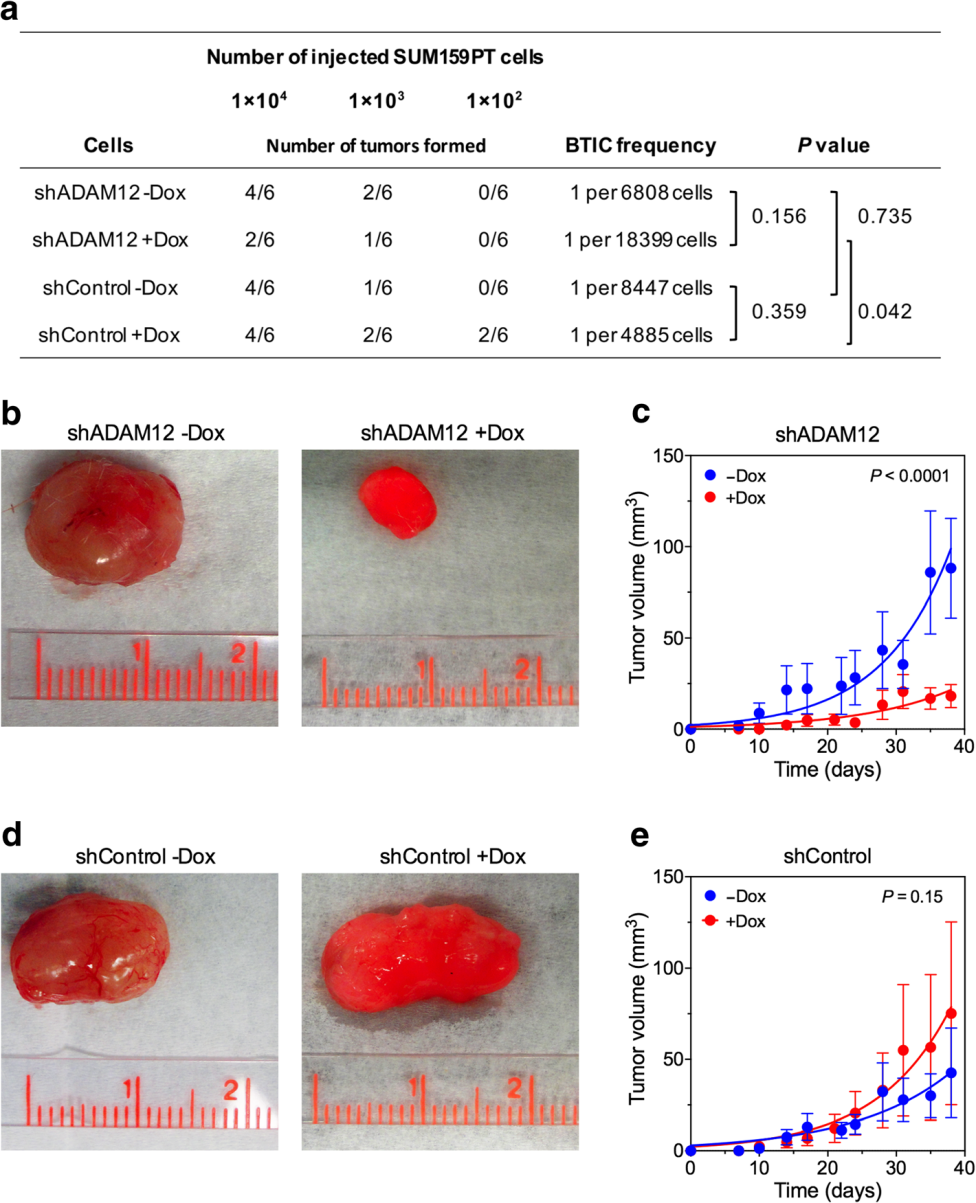
ADAM12 knockdown reduces tumor formation in NOD-SCID mice after orthotopic cell transplantation. **a** Tumor incidence in the limiting dilution transplantation assay. SUM159PT_shADAM12 and SUM159PT_shControl cells were transplanted in the fourth mammary fat pad of NOD-SCID mice in limiting dilutions (10,000, 1,000, or 100 cells per injection site). Prior to transplantation, cells were pre-treated with doxycycline (Dox; 1 μg/ml) for 4 days, and doxycycline (2 g/kg) was then continually administered in the diet. The presence of palpable tumors was determined 3 weeks after cell transplantation. The frequency of breast tumor initiating cells (BTICs) and *P*-values for the statistical significance between groups were calculated using extreme limiting dilution analysis (ELDA, http://bioinf.wehi.edu.au/software/elda/; ref. [44]). **b**, **d** Representative tumors 38 days after injection of 10^5^ SUM159PT_shADAM12 cells (**b**) or SUM159PT_shControl cells (**d**). **c**, **e** Tumor growth was monitored for 0-38 days. Nonlinear regression curves were fitted using GraphPad software

To examine the role of ADAM12 in tumor initiation in vivo, we performed limiting dilution transplantation experiments. SUM159PT_shADAM12 or SUM159PT_shControl cells, pre-treated with doxycycline or vehicle, were injected into NOD-SCID mice as a 10-fold dilution series from 1x10^4^ to 1x10^2^ cells per mammary gland. Mice injected with doxycycline-treated cells were maintained on a doxycycline-containing diet for continuous expression of shRNA. Mice injected with vehicle-treated cells were maintained on a doxycycline-free diet. After monitoring mice for 21 days for tumor formation, the frequency of CSCs among SUM159PT_ shADAM12 cells without or with doxycycline treatment was estimated as ~1/6,808 and ~1/18,399 cells, respectively, using the ELDA algorithm [44] (*P-*value of the difference between groups 0.156; Fig. 4a). Among SUM159PT_shControl cells, without or with doxycycline treatment, the frequency of CSCs was estimated as ~1/8,447 and ~1/4,885 cells, respectively (*P =* 0.359; Fig. 4a). The difference in CSC frequencies between shADAM12 and shControl cells in doxycycline-treated mice was statistically significant (*P* = 0.042, Fig. 4a).

In another approach, 1x10^5^ cells were injected into mouse mammary glands, and tumor growth rates were monitored for up to 38 days. Tumors formed by SUM159PT_shADAM12 cells in doxycycline-treated mice were significantly smaller than tumors detected in untreated mice (*P* < 0.0001; Fig. 4b, c). There was no statistical difference between the growth rates of tumors formed by SUM159PT_shControl cells in doxycycline-treated and untreated mice (Fig. 4d, e). Collectively, these results suggested that ADAM12 knockdown decreased both tumor initiation and tumor growth in mice in vivo.

### Global changes in gene expression upon ADAM12 knockdown

**Fig. 5:**
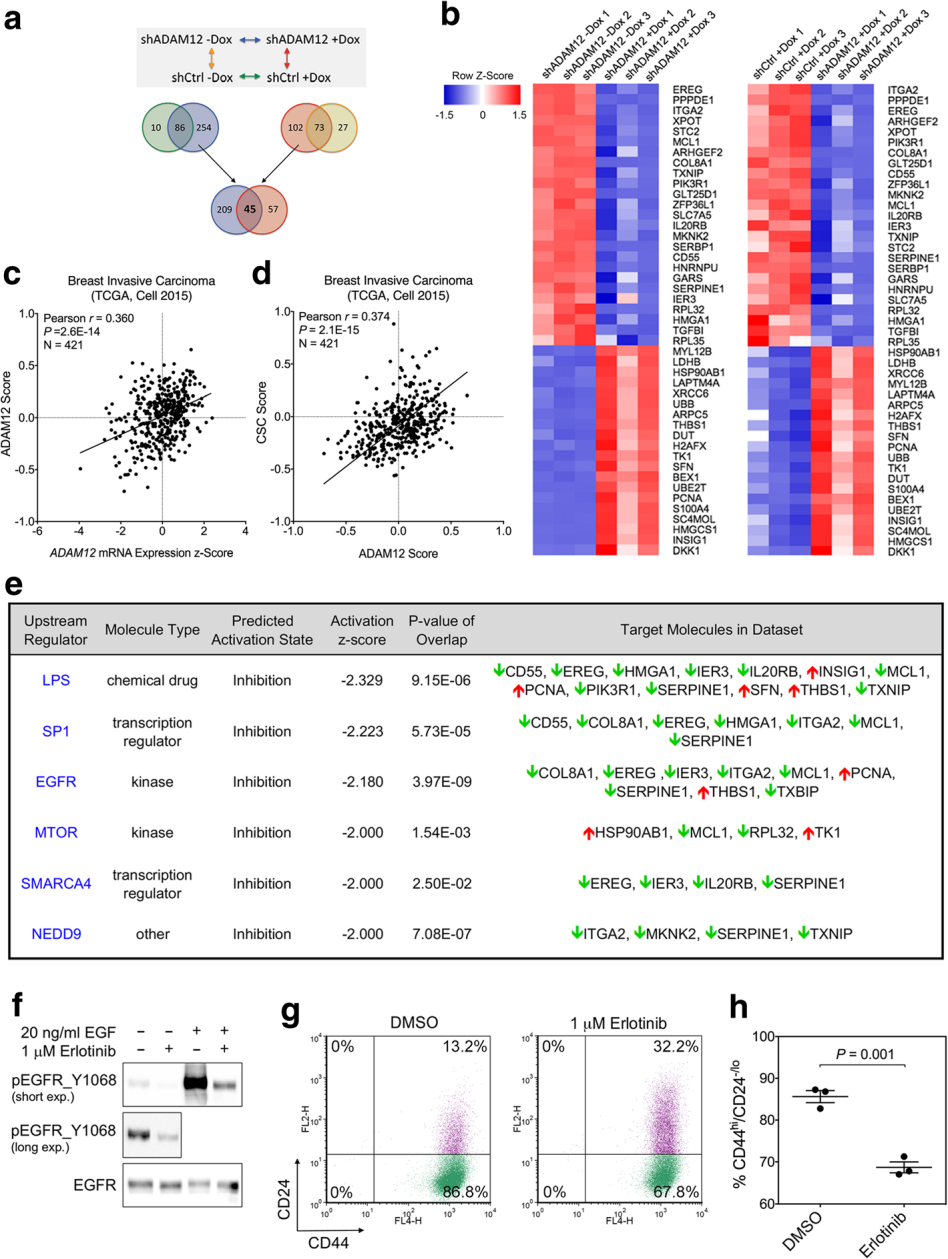
RNA sequencing analysis of the gene expression changes induced by ADAM12 knockdown in SUM159PT cells. **a** Diagram summarizing the design and the outcome of the RNA-Seq experiment. **b** Heatmaps of differentially expressed genes in response to ADAM12 knockdown. Compared are SUM159PT_shADAM12 cells without versus with doxycycline treatment (left) and SUM159PT_ shControl versus SUM159PT_ shADAM12 cells treated with doxycycline (right). **c** ADAM12 gene expression signature score versus *ADAM12* mRNA expression in 421 breast invasive carcinomas from the TCGA database (Cell 2015 dataset). ADAM12 signature scores were generated based on the expression of 45 ADAM12-regulated genes shown in panel b. **d** CSC signature score versus ADAM12 score in 421 breast invasive carcinomas from the TCGA database (Cell 2015 dataset). CSC signature scores were generated based on ref. [22], as described in Methods. **e** Results of the IPA Upstream Regulator analysis. Potential upstream regulators with an overlap *P*‐value < 0.05 and an activation |z score| > 2 are shown. **f** Immunoblot analysis of SUM159PT cells after incubation for 24 h in complete media in the presence or absence of 1 μM erlotinib, an EGFR inhibitor, followed by 30 min treatment with 20 ng/ml EGF, as indicated. **g**, **h** EGFR inhibition mimics the effect of ADAM12 knockdown on the CD44^hi^/CD24^-/lo^ cell population. SUM159PT cells grown in complete medium were treated for 3 days with DMSO or 1 μM erlotinib. **g** CSC-containing CD44^hi^/CD24^-/lo^ population (green) was identified by flow cytometry. **h** Percentage of CD44^hi^/CD24^-/lo^ populations was determined in three independent experiments

To gain insight into global gene expression networks regulated by ADAM12 in breast cancer cells, we performed RNA sequencing of SUM159PT_shADAM12 and SUM159PT_shControl cells, before and after doxycycline treatment. We used two types of comparisons to identify ADAM12-regulated genes (Fig. 5a). First, using a cut-off for | fold change| >1.2 and False Discovery Rate (FDR)-adjusted *P* <0.05, we identified 340 genes that were differentially expressed in SUM159PT_shADAM12 cells with versus without doxycycline treatment and 96 genes that were significantly altered in SUM159PT_shControl cells by doxycycline treatment. Eighty-six genes that were common to these two gene lists were removed from further analysis because their expression was, presumably, affected by doxycycline. Second, we identified 175 genes that were differentially expressed between SUM159PT_shADAM12 versus SUM159PT_shControl cells, in the presence of doxycycline and 100 genes that were differentially expressed in these cells in the absence of doxycycline. Seventy-three genes that were present in both gene sets were removed from further analysis because they presumably represented intrinsic variations between SUM159PT_shADAM12 and SUM159PT_shControl cells. Finally, by combining these two analyses, we generated a high confidence list of 45 genes that were consistently changed in both comparisons and showed the same direction of change (Fig. 5a). Among these, 25 genes were downregulated and 20 genes were upregulated in response to ADAM12 knockdown (Fig. 5b, Additional file 1: Table S5). An ADAM12 gene expression signature score derived from the expression levels of these 45 genes was significantly correlated with *ADAM12* expression in breast tumors from the TCGA database (*P* = 2.6E-14, Fig. 5c). Remarkably, there was also a significant positive correlation between the ADAM12 score and the CSC score in the same tumor dataset (*P* = 2.1E-15, Fig. 5d).

### Biological pathways related to the ADAM12-regulated transcripts

We computed overlaps of the 45 genes altered after ADAM12 knockdown with gene sets from GSEA/MSigDB (http://www.broadinstitute.org/gsea/msigdb). We noted that genes downregulated after ADAM12 knockdown significantly overlapped with genes transiently induced by EGF [45] (*CD55*, *IER3*, *ITGA2*, *MCL1*, *SERPINE1*, *ZFP36L1*; *P* = 1.95E-09), and genes constituting HRAS oncogenic signature [46] (*CD55*, *EREG*, *IER3*, *ITGA2*, *MCL1*; *P =* 2.75E-07) (Additional file 1: Table S6).

To further determine which canonical signaling pathways might have been over-represented among ADAM12-regulated genes, we used Ingenuity Pathway Analysis (IPA, http://www.ingenuity.com). Canonical pathways analysis identified “Signaling by Rho Family GTPases” as the only pathway that was significantly enriched (*P* = 1.25E-04) among genes altered in response to ADAM12 knockdown and had a non-zero (negative) z-score (Additional file 9: Figure S8), indicating that this pathway might have been downregulated after ADAM12 knockdown. To better understand gene expression changes observed in response to ADAM12 knockdown, we applied the IPA Upstream Regulator analysis. The goal of the IPA Upstream Regulator analysis is to identify a cascade of upstream transcriptional regulators that can explain the observed gene expression changes in a user’s dataset [47]. This approach indicated that there was a statistically significant overlap between genes altered after ADAM12 knockdown and the genes regulated by LPS, SP1, EGFR, MTOR, SMARCA4 and NEDD9 transcriptional regulators (Fig. 5e). Among those, an overlap between ADAM12-modulated genes and genes changed in response to EGFR inhibition was the most significant (*P* = 3.97E-09).

### ADAM12 supports the CSC phenotype via modulation of the EGFR pathway

Our investigation of genes changed in response to ADAM12 knockdown in SUM159PT cells using GSEA/MSigDB and IPA gene sets consistently pointed to alterations in the EGFR pathway. Therefore, we tested a hypothesis that ADAM12 supports the CSC phenotype via modulation of the EGFR pathway. First, we examined the effect of erlotinib, a specific inhibitor of EGFR, on SUM159PT cells. Treatment of SUM159PT cells with 1 μM erlotinib inhibited the basal phosphorylation level of EGFR at Y1068, one of the major phosphorylation sites in response to ligand-mediated EGFR activation (Fig. 5f). Importantly, erlotinib elicited a similar effect on the CD44^hi^/CD24^-/lo^ cell population as ADAM12 knockdown did (Fig. 5g, h), suggesting that ADAM12 knockdown might have indeed resulted in inhibition of EGFR signaling.

**Fig. 6:**
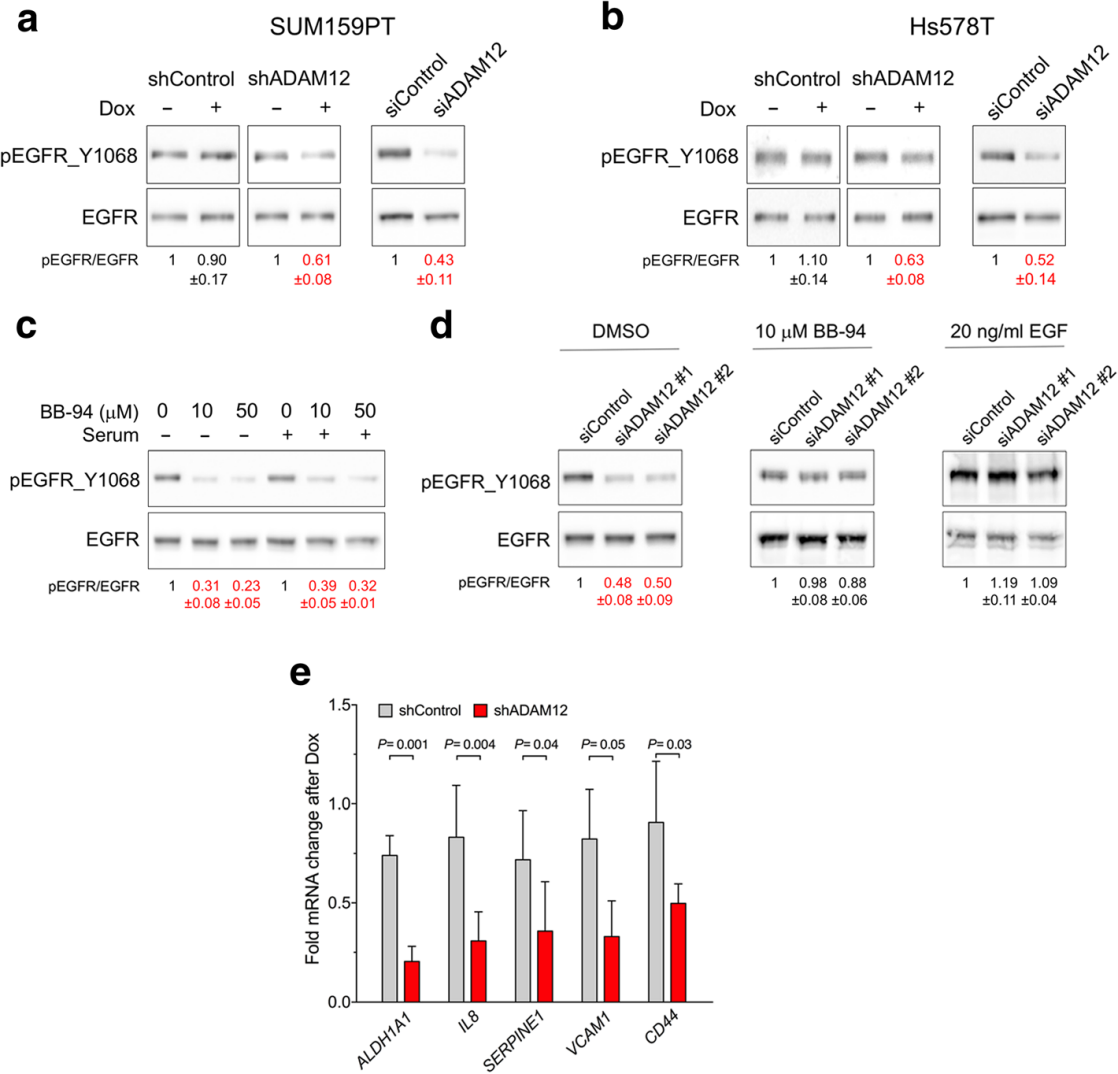
ADAM12 knockdown reduces the basal level of EGFR activation. **a** SUM159PT_shADAM12 or SUM159PT_shControl cells were incubated for 4 days in the presence or absence of 1 μg/ml of doxycycline. Alternatively, SUM159PT cells were transfected with a pool of four control siRNAs (siControl) or a pool of four ADAM12 siRNAs (siADAM12) and analyzed three days later by Western blotting. **b** Hs578T_shADAM12, Hs578T _shControl, or parental Hs578T cells were treated and analyzed as in panel **a. c** The effect of batimastat (BB-94) on the basal activation level of EGFR. SUM159PT cells were incubated for 24 h with 0, 10 μM, or 50 μM BB-94, in the absence or presence of serum, as indicated. **d** The effect of ADAM12 knockdown on the activation of EGFR is abolished by BB-94 or exogenous EGF. SUM159PT cells were transfected with control siRNAs or with two individual ADAM12 siRNAs. Two days after transfection, cells were incubated in complete media supplemented with 10 μM BB-94 for additional 24 h. Alternatively, three days after transfection, cells were incubated for 30 min with 20 ng/ml EGF. In **a**-**d**, representative blots are shown and relative changes in pEGFR/EGFR (means ± SEM; n = 3) are indicated; values significantly lower than 1 (*P* < 0.05) are shown in red. **e** qRT-PCR analysis of the indicated transcripts in SUM159PT_shADAM12 or SUM159PT_shControl cells incubated for 4 days in the presence or absence of 1 μg/ml of doxycycline. Expression was normalized to $\beta$-ACTIN and is shown as fold change after doxycycline treatment; 3≤n≤5

Next, we examined the effect of ADAM12 knockdown on the basal activation level of EGFR. We observed that incubation of SUM159PT_shADAM12 cells, but not SUM159PT_shControl cells, with doxycycline, or transfection of SUM159PT cells with pooled ADAM12 siRNAs decreased the level of EGFR phosphorylation at Y1068 by ~40% and ~55%, respectively (Fig. 6a). Similar results were obtained for Hs578T cells, although the effects were somewhat more modest than in SUM159PT cells (Fig. 6b). Thus, ADAM12 is a positive regulator of EGFR in both cell lines.

Activation of EGFR at the basal level and its susceptibility to inhibition by erlotinib suggested the presence of EGF-like ligands in culture medium. These ligands might have been endogenously expressed by cells and released from the cell surface by ADAM12 and, possibly, other ADAM proteases. Alternatively, low concentrations of EGF-like growth factors might be present in the serum used to supplement culture media. To discriminate between these two possibilities, cells were incubated for 24 h with batimastat (BB-94), a metalloprotease inhibitor, in serum-free medium or in complete medium containing serum. Regardless of the presence or absence of serum, treatment with BB-94 decreased the phosphorylation level of EGFR by 70-80% (Fig. 6c). These results indicated that the basal activation of EGFR was achieved through interactions with endogenous EGF-like ligands that were released from cells via ADAM-mediated cleavage and that were acting in an autocrine/paracrine manner.

To further determine whether ADAM12 played a role in EGFR activation in response to endogenous ligands, we examined the effect of BB-94 or exogenously added EGF on EGFR phosphorylation in cells transfected with control or ADAM12 siRNAs. In these experiments, we used two individual siRNAs from the pool of four siRNAs targeting ADAM12, which diminished cell surface expression of ADAM12 with the highest potency, namely siADAM12#1 and #2, (Additional file 7: Figure S6c). These two individual siRNAs reduced the level of pEGFR_Y1068 by ~50% (Fig. 6d), as seen before with shRNA or pooled siRNAs. Importantly, the effects of siADAM12 #1 and siADAM12 #2 on pEGFR_Y1068 were significantly reduced or eliminated when cells were further incubated with 10 μM BB-94 or 20 ng/ml of EGF (Fig. 6d). These results supported a model in which ADAM12 sustained the basal level of activation of EGFR by mediating the release of endogenous EGF-like ligands.

Since claudin-low tumors have elevated activities of the EGFR pathway [20], we next asked whether reduced activation of EGFR in ADAM12-deficent cells might directly impact the expression of genes associated with claudin-low tumors and cell lines. Indeed, we observed that the expression of several claudin-low-enriched transcripts, *ALDH1A1*, *IL8*, *SERPINE1*, *VCAM1*, and *CD44* [19], was decreased in doxycycline-treated SUM159PT_shADAM12 cells versus SUM159PT_shControl cells (Fig. 6e). These results underscore the role of ADAM12 in promoting the claudin-low phenotype, which is associated with the CSC features.

**Fig. 7:**
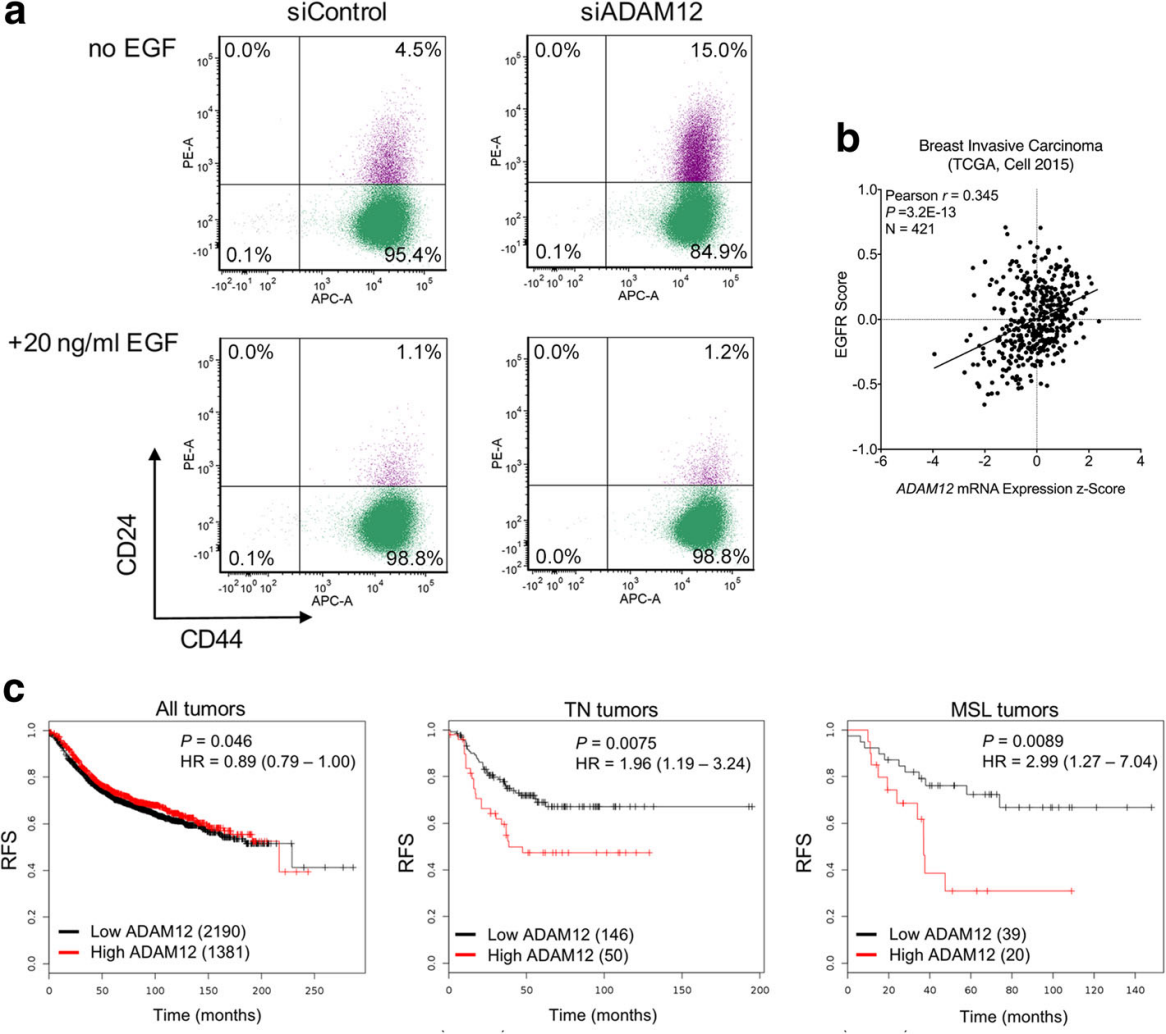
ADAM12 supports the CSC phenotype via modulation of the EGFR pathway. **a** Activation of EGFR by exogenous EGF bypasses the effect of ADAM12 knockdown on the reduction of CD44^hi^/CD24^-/lo^ cell population. SUM159PT cells were transfected with a pool of four control siRNAs (siControl) or a pool of four ADAM12 siRNAs (siADAM12). After 24 h, cells were incubated with or without 20 ng/ml EGF in complete media for additional 48 h and analyzed for CD44 and CD24 expression. **b** EGFR responsive gene signature score versus *ADAM12* mRNA expression in 421 breast invasive carcinomas from the TCGA database (Cell 2015 dataset). EGFR signature scores were calculated based on ref. [37]. **c** Relapse-free survival (RFS) rates for breast cancer patients stratified by *ADAM12* expression levels were estimated using Kaplan-Meier Plotter (http://kmplot.com/analysis/) [39]. Shown are all tumors, triple-negative (TN) tumors, or tumors classified as mesenchymal stem-like (MSL). Hazard ratio (HR), 95% confidence interval, *P*-values, and numbers of patients are also included

To determine whether modulation of the EGFR pathway by ADAM12 is prerequisite for the ADAM12-mediated effect on CSC features, cells were transfected with siADAM12, treated for two days with exogenous EGF, and the cell surface expression of CD44/CD24 markers was evaluated by flow cytometry. Importantly, while EGF increased the pool of CD44^hi^/CD24^-/lo^ cells, as expected, ADAM12 knockdown did not have any effect on CD44 or CD24 expression in the presence of EGF (Fig. 7a). These results suggested that downregulation of EGFR activation by ADAM12 knockdown was required for a reduction of CSC-containing CD44^hi^/CD24^-/lo^ cell population.

We further explored the relationships between *ADAM12* expression and the EGFR-responsive gene signature scores in 421 tumors from the TCGA database. The EGFR scores were calculated based on the gene expression profiling of MCF-7 breast cancer cells overexpressing EGFR [37]. We observed positive correlations between *ADAM12* and the EGFR score (*P* = 3.2E-13, Fig. 7b). Importantly, *ADAM12* expression itself was not significantly changed by manipulations of EGFR in MCF-7 cells [37].

Based on our results, we propose a model where ADAM12 contributes to the activation of EGFR by releasing EGF-like ligands from the cell surface, and ultimately to the expansion of CSC populations. As the EGFR signaling pathway is frequently activated in TNBC, ADAM12-mediated increase of EGFR activity should be the most relevant in this type of cancer. Our model is further supported by the fact that high expression of the *ADAM12* gene correlates with poor prognosis in 196 patients with TNBC and, most importantly, in 59 patients with the MSL subtype of TNBC, but not in a general breast cancer patient population (Fig. 7c, assessed by the Kaplan-Meier Plotter online tool, ref. [39]).

## Discussion

The EGFR pathway activation has been frequently observed in claudin-low TNBCs and cell lines bearing CSC-like features [20], and several reports indicated that EGFR is a positive regulator of CSCs. For example, stimulation of the EGFR pathway promoted mammosphere formation by normal breast stem cells and by ductal carcinoma in situ (DCIS)-derived epithelial cells [48]. Recently, it has been reported that cetuximab, a monoclonal anti-EGFR antibody, reduced mammosphere formation and CSC populations in breast cancer cells in vitro and potentiated the effect of Ixabepilone, a new generation microtubule-stabilizing agent, in treating orthotopic TNBC xenografts [49]. Furthermore, in A431 epidermoid cancer cells, treatment with cetuximab upregulated the expression of the epithelial markers E-cadherin and occludin, downregulated the epithelial transcriptional repressors Zeb, Snail, and Slug, and reduced the CD44^hi^/CD24^-/lo^ phenotype [50]. EGFR activation also promoted acquisition of CSC properties in head and neck squamous cell carcinoma [51, 52] and in nasopharyngeal carcinoma [53], pointing to a more general function of EGFR in CSC biology.

The postulated mechanisms by which EGFR promotes the CSC features in TNBC cells include the activation of MEK/ERK signaling [54], the STAT3 pathway [21], and/or autophagy [49]. For example, blocking ERK activation in claudin-low cell lines by MEK inhibitors or by forced expression of DUSP4, dual specificity phosphatase-4 that is a negative regulator of ERK, has been shown to reduce the CD44^hi^/CD24^-/lo^ populations in vitro and to diminish tumor initiating populations in vivo [54]. STAT3, which is another downstream effector of EGFR, has been recently reported to be preferentially activated in tumor-initiating cells/CSCs in claudin-low breast cancer [21], raising a possibility that it might, at least partially, mediate the downstream effects of EGFR on CSC properties of claudin-low cells.

EGFR is activated by soluble ligands that are synthesized as transmembrane precursors and need to be released from the cell surface by ADAM proteases [5, 55]. In many cell types and tissues, ADAM17 or ADAM10 act as dedicated and robust EGFR ligand “sheddases” [1, 10]. Likewise, it has been postulated that ADAM17 is the main ADAM responsible for EGFR ligand cleavage and activation of EGFR in breast cancer [56–58]. However, other ADAMs, including ADAM12, may mediate the release of soluble EGFR ligands as well. For example, ADAM12 was shown to act as a sheddase for heparin-binding EGF-like growth factor (HB-EGF) during cardiac hypertrophy [59] and under hypoxia in head and neck, lung, and pancreatic cancer cells, leading to the formation of invadopodia and increasing cancer cell invasion [60]. Whether ADAM12 activates EGFR in breast cancer cells and, in particular, whether ADAM12-mediated EGFR activation promotes the acquisition of the breast CSC phenotype, has not been sufficiently explored.

In this report, we have identified ADAM12 as a modifier of the EGFR activation in claudin-low TNBC. In existing microarray datasets, *ADAM12* mRNA levels were the highest in claudin-low and MSL TNBCs, and they strongly correlated with the EMT hallmark gene signature. Among breast cancer cell lines, claudin-low cells expressed the highest levels of ADAM12. Down-regulation of ADAM12 decreased the basal activation levels of EGFR, diminished the expression of claudin-low transcripts, reduced the CSC phenotype in vitro, and decreased the tumorigenic potential of engrafted cells in mice in vivo. In addition, *ADAM12* mRNA expression was strongly correlated with the EGFR activation score in 421 breast invasive cancers. Our previous analysis of two clinical datasets showed that high expression of ADAM12 was predictive of resistance to neoadjuvant chemotherapy in ER-negative breast cancer [18]. This is fully consistent with *ADAM12* being an EMT- and CSC-related gene, as both EMT and CSCs contribute to drug resistance [61, 62]. Finally, in a dataset of 196 TNBCs and 59 MSL TNBCs analyzed here, and previously in 53 TNBC patients without systemic treatment [15, 16], high ADAM12 expression was associated with decreased metastasis-free survival times.

Current treatments of TNBC rely mainly on chemotherapy, as there are no targeted therapies specifically approved for this type of breast cancer [63]. EGFR is expressed in 60-70% of TNBCs [64, 65], raising an early hope for EGFR-targeted therapies in TNBC. Two completed clinical trials investigated the therapeutic potential of cetuximab, as a single agent or in addition to cisplatin chemotherapy, in unselected TNBC patients with metastatic disease [66, 67]. While the results have been disappointing and there was no significant effect on progression-free or overall patient survival, it is becoming clear that EGFR expression alone does not necessarily indicate tumor cell dependence on EGFR signaling and further molecular stratifications and patient selections are needed in future trials [63, 68, 69]. We propose that ADAM12 may be an important biomarker in identifying TNBCs with over-activation of the EGFR pathway and may help select patients that would better respond to EGFR inhibitors. In addition, further detailed studies of ADAM12-mediated regulation of the EGFR pathway should establish whether ADAM12 itself may be a suitable target for CSC-like populations in claudin-low TNBC.

## Conclusions

ADAM12 supports the CSC phenotype in claudin-low breast cancer cells via modulation of the EGFR activation. Therefore, ADAM12 may be an important biomarker in identifying TNBCs that might respond to EGFR inhibition.

## Additional files

Additional file 1: Table S1. Source and characteristics of cell lines used in this study. **Table S2.** Differential expression of ADAM12 in triple-negative breast cancer subtypes, based on Lehmann et al. [36]. **Table S3.** The list of top 40 genes correlated with ADAM12 expression in breast invasive carcinomas from the TCGA database. **Table S4.** The list of hallmark GSEA/MSigDB gene signatures enriched in the 40 genes from Table S2. **Table S5.** The list of 45 genes changed by ADAM12 knockdown in SUM159PT cells. **Table S6.** Overlaps between ADAM12-regulated genes and gene sets in GSEA/MSigDB. **Table S7.** qRT-PCR primer sequences. (PDF 76 kb)
Additional file 2: Figure S1.
*ADAM12* mRNA is upregulated in claudin-low breast cancers. a Heatmap showing *ADAM12* mRNA expression levels in 295 breast cancer patients from the Netherlands Cancer Institute (NKI) database [31]. Molecular subtypes of tumors are: B, basal; CL, claudin-low; HER2+, HER2-enriched; LumA, luminal A; LumB, luminal B; NL, normal-like. b Expression levels of *ADAM12* in 508 breast cancer patients from The Cancer Genome Atlas (TCGA, Nature 2012 dataset) [33]. (TIF 4437 kb)
Additional file 3: Figure S2. Histogram analysis of a representative flow cytometry data showing the effect of 10 nM paclitaxel on ADAM12 expression in SUM159PT cells. Cells were treated for 6 days with DMSO (control) or with 10 nM paclitaxel (PTX), and then allowed to recover for 6 days without PTX. Live cells were stained with anti-ADAM12 antibody and propidium iodide (PI); only viable (PI-negative) cells were included in the analysis. To further identify ADAM12-positive cells, histogram subtraction of partially overlapping sample (anti-ADAM12 Ab) and control (isotype control Ab) histograms was performed using the FCS Express 4 software. Median fluorescence intensity of the resulting curves and the 5th and 95th percentiles for DMSO- and PTX-treated cells are shown. (TIF 3184 kb)
Additional file 4: Figure S3. Sorting ADAM12^hi^ and ADAM12^med^ subpopulations of SUM159PT cells. Live cells were stained with anti-ADAM12 antibody and propidium iodide (PI). A population of ~1.5% viable (PI-negative) cells with the highest expression of ADAM12, ADAM12^hi^, and a population with medium expression levels of ADAM12, ADAM12^med^, corresponding to the middle ~75% of all viable cells, were sorted. A subsequent post-sort flow cytometry and qRT-PCR analyses confirmed an enrichment of ADAM12^hi^ and ADAM12^med^ cells within sorted populations. (TIF 221 kb)
Additional file 5: Figure S4. ADAM12^hi^ subpopulation of SUM159PT cells is enriched for cancer stem cell-like features. a Growth rates of sorted ADAM12^hi^ and ADAM12^med^ populations of SUM159PT cells. Sorted cells were plated in 96-well plates in triplicates and the relative numbers of live cells were determined 24 h -72 h later using the CellTiter-Glo viability assay. Graph represents mean values ± SEM from 2 independent experiments, as well as fitted exponential growth curves with 95% confidence interval. b Mammosphere formation by ADAM12^hi^ and ADAM12^med^ populations. Sorted cells were plated in ultra-low attachment plates in mammosphere medium containing 1% methylcellulose. After 10 days, primary (1^o^) mammospheres were collected, trypsinized, and single cell suspensions were seeded for secondary (2^o^) mammosphere formation. Mammospheres were visualized by phase contrast imaging, and the numbers of spheres with diameters > 50 μm were counted using ImageJ. The results are shown as means ± SEM obtained from 3 independent experiments. c-e, Relative expression of cancer stem cell markers (c), epithelial-to-mesenchymal transition markers (d), and cancer drug resistance genes (e) in ADAM12^hi^ versus ADAM12^med^ subpopulations of SUM159PT cells. Sorted cell populations were analyzed by qRT-profiling using RT^2^ Profiler PCR Arrays (Qiagen). Genes included in the arrays are shown in black, the data for ADAM12 is shown in red. (TIF 1278 kb)
Additional file 6: Figure S5. Doxycycline-inducible ADAM12 knockdown in SUM159PT cells. a Diagram of the doxycycline-inducible lentiviral shRNA construct targeting ADAM12. 5’-LTR, 5’-long terminal repeat; TRE, tetracycline-inducible promoter; tRFP, turbo red fluorescent protein; miR30-5’ and shADAM12, micro-RNA-30-adapted shRNA targeting ADAM12; Ubc, human ubiquitin C promoter; rtTA3, reverse tetracycline-transactivator 3; IRES, internal ribosomal entry site; Puro, puromycin resistance gene; 3’-LTR, 3’-long terminal repeat. b, c, d Validation of ADAM12 knockdown. Stably transduced SUM159PT_shADAM12 and SUM159PT_shControl cells were incubated for 4 days with or without 1 μg/ml of doxycycline. b ADAM12 mRNA levels were quantified by qRT-PCR. c ADAM12 protein levels were determined by Western blotting after glycoprotein enrichment on concanavalin A agarose. EGFR serves as a loading control for glycoprotein-enriched fractions. Arrow, the nascent full length ADAM12; arrowhead, the processed form lacking the N-terminal pro-domain. d Cell surface expression of ADAM12 was examined by flow cytometry. (TIF 1149 kb)
 Additional file 7: Figure S6. ADAM12 knockdown using siRNAs. a SUM159PT cells were transfected with a pool of four siRNAs targeting ADAM12 or a pool of control siRNAs. b Hs578T cells were transfected with a pool of four siRNAs targeting ADAM12 or with a pool of control siRNAs. c SUM159PT cells were transfected with individual siRNAs, as indicated. Cell surface expression of ADAM12 was evaluated by flow cytometry. (TIF 556 kb)
Additional file 8: Figure S7. Inducible ADAM12 knockdown in SUM159PT cells increases cell surface expression of CD24. SUM159PT_shADAM12 (a) or SUM159_shControl cells (b) were incubated for 4 days with or without 1 μg/ml of doxycycline. Cell surface expression was evaluated by flow cytometry. Percentages of CD24^+^ cells stained with FITC-conjugated anti-CD24 antibody, but not with isotype control antibody, are indicated. (TIF 706 kb)
Additional file 9: Figure S8. Canonical pathways identified by IPA based on differentially expressed genes in response to ADAM12 knockdown. *P*-values for the canonical pathways are calculated by Fisher's exact test, right-tailed. The orange and blue colored bars indicate predicted pathway activation, or predicted inhibition, respectively (z-score). (TIF 572 kb)

## Abbreviations

ADAM: A disintegrin and metalloprotease
AEBSF: 4-(2-aminoetyl)benzenesulfonyl fluoride hydrochloride
ALDH: Aldehyde dehydrogenase
APC: Allophycocyanin
BAA^-^: BODIPY-aminoacetate
BSA: Bovine serum albumin
BTICs: Breast tumor initiating cells
CSCs: Cancer stem cells
DCIS: Ductal carcinoma in situ
DEAB: Diethylamino-benzaldehyde
DMEM: Dulbecco’s modified Eagle’s medium
Dox: Doxycycline
DPBS: Dulbecco’s phosphate-buffered saline
DUSP4: Dual specificity phosphatase-4
EDGE: Empirical analysis of digital gene expression tool
EDTA: Ethylenediaminetetraacetic acid
EGF: Epidermal growth factor
EGFR: Epidermal growth factor receptor
ELDA: Extreme limiting dilution analysis
ELISA: Enzyme linked immunosorbent assay
EMT: Epithelial-to-mesenchymal transition
ER: Estrogen receptor
ERK: Extracellular signal-regulated kinase 2
FACS: Fluorescence-activated cell sorting
FBS: Fetal bovine serum
FDR: False discovery rate
FGF: Fibroblast growth factor
FITC: Fluorescein isothiocyanate
FSC: Forward scatter
GEO: Gene Expression Omnibus
GSEA/MSigDB: Gene set enrichment analysis/molecular signature database
HEPES: 4-(2-hydroxyethyl)-1-piperazineethanesulfonic acid
HER2: Human epidermal growth factor receptor 2
HR: Hazard ratio
HRP: Horseradish peroxidase
IPA: Ingenuity pathway analysis
MEK: Mitogen-activated protein kinase kinase
MEMB: Mammary epithelial basal medium
NGS: Next-generation sequencing
NOD-SCID: Non-obese diabetic severe combined immunodeficient
PE: Phycoerythrin
PI: Propidium iodide
PR: Progesterone receptor
PTX: Paclitaxel
qRT-PCR: Quantitative real time polymerase chain reaction
RFP: Red fluorescent protein
RFS: Relapse-free survival
RPKM: Reads per kilobase of transcript per million mapped reads
SSC: Side cell scatter
STAT3: Signal transducer and activator of transcription 3
STR: Short tandem repeat
TCGA: The cancer genome atlas
TGFβ: Transforming growth factor β
TNBC: Triple negative breast cancer
