# Supplementary material for: Metalloprotease-disintegrin ADAM12 actively promotes the stem cell-like phenotype in claudin-low breast cancer
Source: Mol Cancer. 2017 Feb 1;16:32. doi: 10.1186/s12943-017-0599-6 (PMC5288940; doi:10.1186/s12943-017-0599-6)
Supplement: Additional file 1: Table S1. — Source and characteristics of cell lines used in this study. Table S2. Differential expression of ADAM12 in triple-negative breast cancer subtypes, based on Lehmann et al. [36]. Table S3. The list of top 40 genes correlated with ADAM12 expression in breast invasive carcinomas from the TCGA database. Table S4. The list of hallmark GSEA/MSigDB gene signatures enriched in the 40 genes from Table S2. Table S5. The list of 45 genes changed by ADAM12 knockdown in SUM159PT cells. Table S6. Overlaps between ADAM12-regulated genes and gene sets in GSEA/MSigDB. Table S7. qRT-PCR primer sequences. (PDF 76 kb) [file 12943_2017_599_MOESM1_ESM.pdf]

Table S1. Source and characteristics of cell lines used in this study.

**Cell line**	**Cell source**	**Tumor pathology**	**Cell morphology**	**Molecular classification (1)**	**Intrinsic subtype (2,3)**	**ER**	**PR**	**HER2**	**TP53 (4,5)**
BT549	PBr	IDC	Spindle	Basal B	Claudin-low	-	-	-	M, ++
Hs578T	PBr	IDC	Spindle	Basal B	Claudin-low	-	-	-	M, ++
MCF-7	PE	IDC	Epithelial	Luminal	Luminal	+	+	-	WT, +
SUM102PT	PBr	IDC	Spindle	Basal B	Basal-like	-	-	+	WT, +
SUM1315MO2	Sk	IDC	Spindle	Basal B	Claudin-low	-	-	+	M, ++
SUM149PT	PBr	Inf Duc Ca	Mixed	Basal B	Basal-like	-	-	+	M, ++
SUM159PT	PBr	An Ca	Spindle	Basal B	Claudin-low	-	-	-	M, ++
SUM225CWN	CWN	IDC	Epithelial	Basal A	Luminal	-	-	++	M, ++

Abbreviations: An Ca, anaplastic carcinoma; CWN, chest wall nodule; Inf Duc Ca, inflammatory ductal carcinoma; IDC, invasive ductal carcinoma; PBr, primary breast cancer; PE, pleural effusion; Sk, skin.

ER/PR/HER2/TP53 status: ER/PR positivity, HER2 overexpression, and TP53 protein levels and mutational status (M, mutant protein; WT, wild-type protein) are indicated.

References:

1. Neve et al., Cancer Cell. 2006;10:515-527.
2. Prat et al., Breast Cancer Res. 2010;12:R68.
3. Prat et al., Breast Cancer Res Treat. 2013;142:237-255.
4. Hollestelle et al., Breast Cancer Res Treat 2010, 121:53-64.
5. Barnabas et al., Int J Breast Cancer. 2013:872743.

Table S2. Differential expression of ADAM12 in triple-negative breast cancer subtypes, based on Lehmann *et al.*

Subtype	Regulation	*P*-value
Mesenchymal (M)	-	
Mesenchymal stem-like (MSL)	Up	5.19E-08
Basal-like 1 (BL1)	-	
Basal-like 2 (BL2)	-	
Immunomodulatory (IM)	Down	5.19E-08
Luminal androgen receptor (LAR)	-	

Table S3. The list of top 40 genes correlated with ADAM12 expression in breast invasive carcinomas from the TCGA database.

Gene Symbol	Pearson Score	Spearman Score	Gene Description	Entrez Gene ID
FAP	0.9	0.9	fibroblast activation protein, alpha	2191
COL5A1	0.89	0.89	collagen, type V, alpha 1	1289
COL5A2	0.89	0.88	collagen, type V, alpha 2	1290
COL1A2	0.88	0.87	collagen, type I, alpha 2	1278
POSTN	0.88	0.89	periostin, osteoblast specific factor	10631
FBN1	0.87	0.86	fibrillin 1	2200
SPARC	0.87	0.87	secreted protein, acidic, cysteine-rich	6678
THBS2	0.87	0.87	thrombospondin 2	7058
VCAN	0.87	0.87	versican	1462
COL6A3	0.86	0.85	collagen, type VI, alpha 3	1293
DACT1	0.86	0.86	dapper, antagonist of beta-catenin, homolog 1	51339
GLT8D2	0.86	0.85	glycosyltransferase 8 domain containing 2	83468
COL1A1	0.85	0.84	collagen, type I, alpha 1	1277
BNC2	0.84	0.85	basonuclin 2	54796
CDH11	0.84	0.83	cadherin 11	1009
COL6A2	0.84	0.83	collagen, type VI, alpha 2	1292
CTSK	0.84	0.84	cathepsin K	1513
LUM	0.84	0.83	lumican	4060
MAGEL2	0.84	0.83	MAGE-like 2	54551
SPON1	0.84	0.83	spondin 1, extracellular matrix protein	10418
MMP2	0.83	0.83	matrix metallopeptidase 2	4313
PPAPDC1A	0.83	0.84	phosphatidic acid phosphatase type 2 domain containing 1A	196051
SRPX2	0.82	0.8	sushi-repeat containing protein, X-linked 2	27286
WISP1	0.82	0.81	WNT1 inducible signaling pathway protein 1	8840
DCN	0.81	0.8	decorin	1634
FSTL1	0.81	0.8	follistatin-like 1	11167
HTRA1	0.81	0.84	HtrA serine peptidase 1	5654
LRRC15	0.81	0.8	leucine rich repeat containing 15	131578
NID2	0.81	0.81	nidogen 2	22795
P4HA3	0.81	0.82	prolyl 4-hydroxylase, alpha polypeptide III	283208
PCOLCE	0.81	0.8	procollagen C-endopeptidase enhancer	5118
COL6A1	0.8	0.78	collagen, type VI, alpha 1	1291
FN1	0.8	0.8	fibronectin 1	2335
PRRX1	0.8	0.79	paired related homeobox 1	5396
SERPINF1	0.8	0.78	serpin peptidase inhibitor, clade F, member 1	5176
SFRP2	0.8	0.79	secreted frizzled-related protein 2	6423
THY1	0.8	0.79	Thy-1 cell surface antigen	7070
AEBP1	0.79	0.78	AE binding protein 1	165
RARRES2	0.79	0.77	retinoic acid receptor responder 2	5919
SGCD	0.79	0.78	sarcoglycan, delta	6444

Table S4. The list of hallmark GSEA/MSigDB gene signatures enriched in the 40 genes from Table S2.

Gene Set Name	# Genes in Gene Set (K)	# Genes in Overlap (k)	k/K	*P*-value	FDR q-value	Overlap
HALLMARK_EPITHELIAL_MESENCHYMAL_TRANSITION	200	25	0.125	7.43E-50	3.71E-48	CDH11, COL1A1, COL1A2, COL5A1, COL5A2, COL6A2, COL6A3, DCN, FAP, FBN1, FN1, FSTL1, HTRA1, LRRC15, LUM, MMP2, NID2, PCOLCE, POSTN, PRRX1, SGCD, SPARC, THBS2, THY1, VCAN
HALLMARK_ANGIOGENESIS	36	5	0.1389	1.42E-10	3.56E-09	COL5A2, FSTL1, LUM, POSTN, VCAN
HALLMARK_COAGULATION	138	6	0.0435	2.32E-09	3.86E-08	CTSK, FBN1, FN1, HTRA1, MMP2, SPARC
HALLMARK_MYOGENESIS	200	6	0.03	2.14E-08	2.67E-07	AEBP1, COL1A1, COL6A2, COL6A3, SGCD, SPARC
HALLMARK_APICAL_JUNCTION	200	5	0.025	8.63E-07	8.63E-06	CDH11, FBN1, MMP2, THY1, VCAN
HALLMARK_UV_RESPONSE_DN	144	3	0.0208	2.73E-04	2.28E-03	COL1A1, COL1A2, COL5A2
HALLMARK_APOPTOSIS	161	3	0.0186	3.79E-04	2.71E-03	DCN, LUM, MMP2
HALLMARK_GLYCOLYSIS	200	3	0.015	7.12E-04	4.45E-03	VCAN, DCN, COL5A1

Table S5. The list of 45 genes changed by ADAM12 knockdown in SUM159PT cells.

	shADAM12 +Dox vs -Dox		shADAM12 +Dox vs shCtrl +Dox			
**Feature ID**	**Fold change**	**FDR corrected *P*-value**	**Fold change**	**FDR corrected *P*-value**	**Gene Description**	**Entrez Gene ID**
EREG	-3.24	1.48E-15	-2.30	1.17E-05	Epiregulin	2069
PPPDE1	-2.91	9.91E-06	-2.31	1.47E-02	PPPDE peptidase domain containing 1	51029
ITGA2	-2.88	1.99E-06	-2.51	4.45E-04	Integrin, alpha 2	3673
XPOT	-2.22	5.55E-15	-1.86	1.56E-07	Exportin, tRNA	11260
STC2	-2.15	2.67E-17	-1.51	5.73E-03	Stanniocalcin 2	8614
MCL1	-2.06	6.03E-26	-1.67	1.79E-10	Myeloid cell leukemia sequence 1 (BCL2-related)	4170
ARHGEF2	-1.88	2.46E-04	-1.90	3.41E-04	Rho/Rac guanine nucleotide exchange factor (GEF) 2	9181
COL8A1	-1.88	9.58E-07	-1.79	2.88E-05	Collagen, type VIII, alpha 1	1295
TXNIP	-1.82	8.36E-18	-1.53	6.99E-04	Thioredoxin interacting protein	10628
PIK3R1	-1.81	9.23E-03	-1.84	9.44E-03	Phosphoinositide-3-kinase, regulatory subunit 1 alpha	5295
GLT25D1	-1.80	8.80E-06	-1.75	7.80E-05	Glycosyltransferase 25 domain containing 1	79709
ZFP36L1	-1.78	3.28E-06	-1.72	6.63E-05	Zinc finger protein 36, C3H type-like 1	677
SLC7A5	-1.74	7.57E-15	-1.36	1.63E-02	Solute carrier family 7, member 5	8140
IL20RB	-1.66	1.47E-04	-1.64	6.46E-04	Interleukin 20 receptor beta	53833
MKNK2	-1.65	1.04E-02	-1.70	5.38E-03	MAP kinase interacting serine/threonine kinase 2	2872
SERBP1	-1.59	9.85E-06	-1.45	7.12E-03	SERPINE1 mRNA binding protein 1	26135
CD55	-1.58	4.32E-03	-1.74	8.92E-05	CD55 molecule, decay accelerating factor for complement	1604
HNRNPU	-1.51	2.52E-04	-1.39	4.36E-02	Heterogeneous nuclear ribonucleoprotein U	3192
GARS	-1.47	2.59E-06	-1.44	6.26E-04	Glycyl-tRNA synthetase	2617
SERPINE1	-1.40	0.00E+00	-1.48	0.00E+00	Serpin peptidase inhibitor, clade E, member 1	5054
IER3	-1.33	4.43E-02	-1.56	1.49E-05	Immediate early response 3	8870
RPL32	-1.27	1.44E-02	-1.28	1.38E-02	Ribosomal protein L32	6161
HMGA1	-1.27	1.72E-03	-1.27	6.36E-03	High mobility group AT-hook 1	3159
TGFBI	-1.24	2.10E-06	-1.24	1.74E-06	Transforming growth factor, beta-induced	7045
RPL35	-1.18	2.30E-02	-1.22	3.54E-02	Ribosomal protein L35	11224
MYL12B	1.21	2.34E-02	1.38	4.01E-07	Myosin, light chain 12B, regulatory	103910
LDHB	1.24	4.34E-03	1.29	2.64E-04	Lactate dehydrogenase B	3945
HSP90AB1	1.25	6.47E-08	1.22	7.13E-03	Heat shock protein 90kDa alpha (cytosolic), class B member 1	3326
LAPTM4A	1.46	4.83E-03	1.45	1.14E-02	Lysosomal protein transmembrane 4 alpha	9741
XRCC6	1.49	2.27E-06	1.35	4.88E-03	X-ray repair complementing defective repair in Chinese hamster cells 6	2547
UBB	1.66	3.78E-05	1.77	2.85E-06	Ubiquitin B	7314
ARPC5	1.75	1.46E-04	1.54	1.87E-02	Actin related protein 2/3 complex, subunit 5	10092
THBS1	1.76	3.16E-04	1.60	1.14E-02	Thrombospondin 1	7057
DUT	1.79	4.79E-02	1.96	1.99E-02	Deoxyuridine triphosphatase	1854
H2AFX	1.81	3.67E-06	1.57	4.69E-02	H2A histone family, member X	3014
TK1	1.94	8.80E-06	1.85	8.21E-05	Thymidine kinase 1, soluble	7083
SFN	2.15	7.90E-07	1.62	4.75E-02	Stratifin	2810
BEX1	2.34	5.93E-05	2.00	2.88E-03	Brain expressed, X-linked 1	55859
UBE2T	2.47	1.13E-08	2.08	9.24E-06	Ubiquitin-conjugating enzyme E2T (putative)	29089
PCNA	2.50	4.63E-14	1.67	1.12E-03	Proliferating cell nuclear antigen	5111
S100A4	2.59	2.83E-07	1.96	1.38E-03	S100 calcium binding protein A4	6275
SC4MOL	4.69	4.90E-09	2.51	1.14E-03	Methylsterol monooxygenase 1	6307
HMGCS1	5.45	2.54E-08	2.86	5.81E-04	3-hydroxy-3-methylglutaryl-CoA synthase 1 (soluble)	3157
INSIG1	6.74	3.02E-11	2.34	4.87E-03	Insulin induced gene 1	3638
DKK1	8.61	1.48E-05	5.32	7.01E-04	Dickkopf homolog 1	22943

Table S6. Overlaps between ADAM12-regulated genes and genesets in GSEA/MSigDB

The list of GSEA gene signatures enriched among genes **down-regulated** by ADAM12 KD

Collection(s):	C6, CGP
# overlaps shown:	20
# genesets in collections:	3584
# genes in comparison (n):	25
# genes in universe (N):	45956

Gene Set Name	# Genes in Gene Set (K)	Description	# Genes in Overlap (k)	k/K	*P*-value	FDR q-value	Overlap
JIANG_HYPOXIA_NORMAL	311	Genes up-regulated in RPTEC cells (normal kidney) by h	8	0.0257	3.93E-12	1.41E-08	ARHGEF2, HNRNPU, MCL1, SERBP1, SERPINE1, STC2, TXNIP, ZFP36L1
ZWANG_CLASS_3_TRANSIENTLY_INDUCED_BY_EGF	222	Class III of genes transiently induced by EGF [GeneID =1	6	0.027	1.95E-09	3.49E-06	CD55, IER3, ITGA2, MCL1, SERPINE1, ZFP36L1
BLUM_RESPONSE_TO_SALIRASIB_UP	245	Selected genes up-regulated in response to the Ras inh	6	0.0245	3.51E-09	4.20E-06	ARHGEF2, CD55, GARS, MKNK2, STC2, XPOT
BUYTAERT_PHOTODYNAMIC_THERAPY_STRESS_UP	811	Genes up-regulated in T24 (bladder cancer) cells in res	8	0.0099	7.54E-09	6.11E-06	ARHGEF2, CD55, EREG, IER3, ITGA2, MKNK2, STC2, TXNIP
KOINUMA_TARGETS_OF_SMAD2_OR_SMAD3	824	Genes with promoters occupied by SMAD2 or SMAD3 [	8	0.0097	8.53E-09	6.11E-06	CD55, IER3, ITGA2, MCL1, SERPINE1, SLC7A5, TGFBI, ZFP36L1
PRAMOONJAGO_SOX4_TARGETS_UP	52	Genes up-regulated in ACC3 cells (adenoid cystic carcin	4	0.0769	1.81E-08	1.08E-05	IER3, SERPINE1, STC2, ZFP36L1
MANALO_HYPOXIA_UP	207	Genes up-regulated in response to both hypoxia and o	5	0.0242	8.72E-08	4.47E-05	IER3, SERPINE1, STC2, TGFBI, TXNIP
KIM_WT1_TARGETS_UP	214	Genes up-regulated in UB27 cells (osteosarcoma) at an	5	0.0234	1.03E-07	4.61E-05	EREG, HMGA1, IER3, SERPINE1, ZFP36L1
DELYS_THYROID_CANCER_UP	443	Genes up-regulated in papillary thyroid carcinoma (PTC	6	0.0135	1.18E-07	4.68E-05	CD55, COL8A1, EREG, IER3, ITGA2, SLC7A5
MIYAGAWA_TARGETS_OF_EWSR1_ETS_FUSIONS_DN	229	Genes commonly down-regulated in UET-13 cells (mes	5	0.0218	1.44E-07	5.16E-05	COL8A1, EREG, HMGA1, SERPINE1, STC2
GARY_CD5_TARGETS_UP	473	Genes up-regulated in Daudi cells (B lymphocytes) stab	6	0.0127	1.73E-07	5.63E-05	CD55, GARS, HMGA1, MKNK2, PPPDE1, ZFP36L1
BILD_HRAS_ONCOGENIC_SIGNATURE	261	Genes selected in supervised analyses to discriminate c	5	0.0192	2.75E-07	8.00E-05	CD55, EREG, IER3, ITGA2, MCL1
CHEN_LVAD_SUPPORT_OF_FAILING_HEART_UP	103	Up-regulated genesin the left ventricle myocardium of	4	0.0388	2.90E-07	8.00E-05	MCL1, PIK3R1, SERPINE1, TXNIP
WANG_SMARCE1_TARGETS_UP	280	Genes up-regulated in BT549 cells (breast cancer) by ex	5	0.0179	3.90E-07	9.97E-05	CD55, COL8A1, ITGA2, TGFBI, TXNIP
BROWNE_HCMV_INFECTION_14HR_DN	298	Genes down-regulated in primary fibroblast cell culture	5	0.0168	5.30E-07	1.25E-04	HNRNPU, ITGA2, SERPINE1, STC2, ZFP36L1
TOOKER_GEMCITABINE_RESISTANCE_DN	122	Down-regulated genes in Calu3 cells (non-small cell lun	4	0.0328	5.73E-07	1.25E-04	CD55, MKNK2, SLC7A5, XPOT
BORCZUK_MALIGNANT_MESOTHELIOMA_UP	305	Genes up-regulated in biphasic (mixed) vs epithelial sul	5	0.0164	5.94E-07	1.25E-04	GARS, HMGA1, PPPDE1, SERBP1, XPOT
MISSIAGLIA_REGULATED_BY_METHYLATION_UP	126	Genes up-regulated in PaCa44 and CFPAC1 cells (pancr	4	0.0317	6.52E-07	1.30E-04	CD55, ITGA2, SERPINE1, TXNIP
LEI_MYB_TARGETS	318	Myb-regulated genes in MCF7 (breast cancer) and lung	5	0.0157	7.29E-07	1.32E-04	MCL1, SERBP1, SERPINE1, SLC7A5, TGFBI
ELVIDGE_HYPOXIA_BY_DMOG_UP	130	Genes up-regulated in MCF7 cells (breast cancer) treat	4	0.0308	7.38E-07	1.32E-04	SERPINE1, STC2, TGFBI, TXNIP

The list of GSEA gene signatures enriched among genes **up-regulated** by ADAM12 KD

Collection(s):	C6, CGP
# overlaps shown:	20
# genesets in collections:	3584
# genes in comparison (n):	20
# genes in universe (N):	45956

Gene Set Name	# Genes in Gene Set (K)	Description	# Genes in Overlap (k)	k/K	*P*-value	FDR q-value	
CASORELLI_ACUTE_PROMYELOCYTIC_LEUKEMIA_DN	663	Genes down-regulated in APL (acute promyeolocytic le	9	0.0136	3.74E-12	1.34E-08	BEX1, HMGCS1, HSP90AB1, INSIG1, LDHB, MSMO1, THBS1, TK1, XRCC6
WANG_RESPONSE_TO_GSK3_INHIBITOR_SB216763_DN	374	Genes down-regulated in RS4;11 cells (MLL, mixed line	7	0.0187	1.58E-10	2.84E-07	BEX1, H2AFX, HMGCS1, INSIG1, MSMO1, TK1, UBE2T
ISHIDA_E2F_TARGETS	53	Genes up-regulated in MEF cells (embryonic fibroblast)	4	0.0755	7.53E-09	9.00E-06	DUT, PCNA, TK1, UBE2T
REN_BOUND_BY_E2F	61	Genes whose promoters were bound by E2F1 and E2F4	4	0.0656	1.34E-08	1.20E-05	DUT, H2AFX, PCNA, TK1
CROONQUIST_NRAS_SIGNALING_DN	72	Genes down-regulated in ANBL-6 cell line (multiple my	4	0.0556	2.63E-08	1.60E-05	DUT, H2AFX, PCNA, TK1
DELYS_THYROID_CANCER_UP	443	Genes up-regulated in papillary thyroid carcinoma (PTC	6	0.0135	2.68E-08	1.60E-05	H2AFX, MSMO1, S100A4, SFN, THBS1, TK1
INGA_TP53_TARGETS	17	Genes whose promoters contain TP53 [GeneID=7157] r	3	0.1765	4.77E-08	2.43E-05	PCNA, SFN, THBS1
DAZARD_RESPONSE_TO_UV_NHEK_UP	244	Genes up-regulated in NHEK cells (normal keratinocyte	5	0.0205	5.88E-08	2.43E-05	H2AFX, HSP90AB1, SFN, TK1, UBB
MORI_IMMATURE_B_LYMPHOCYTE_DN	90	Down-regulated genes in the B lymphocyte developme	4	0.0444	6.50E-08	2.43E-05	DUT, H2AFX, PCNA, XRCC6
KOBAYASHI_EGFR_SIGNALING_24HR_DN	251	Genes down-regulated in H1975 cells (non-small cell lu	5	0.0199	6.77E-08	2.43E-05	DKK1, DUT, H2AFX, PCNA, TK1
GOBERT_OLIGODENDROCYTE_DIFFERENTIATION_UP	570	Genes up-regulated during later stage of differentiatior	6	0.0105	1.19E-07	3.86E-05	DUT, H2AFX, LDHB, S100A4, TK1, UBE2T
BASAKI_YBX1_TARGETS_UP	290	Genes up-regulated in SKOC-3 cells (ovarian cancer) aft	5	0.0172	1.39E-07	4.14E-05	DKK1, H2AFX, SFN, THBS1, TK1
SCHMIDT_POR_TARGETS_IN_LIMB_BUD_UP	26	Genes up-regulated in E12.5 forelimb buds with POR [G	3	0.1154	1.82E-07	5.02E-05	HMGCS1, INSIG1, MSMO1
DUTERTRE_ESTRADIOL_RESPONSE_24HR_UP	324	Genes up-regulated in MCF7 cells (breast cancer) at 24	5	0.0154	2.40E-07	6.15E-05	DUT, H2AFX, PCNA, TK1, UBE2T
GOLDRATH_ANTIGEN_RESPONSE	346	Genes up-regulated at the peak of an antigen response	5	0.0145	3.32E-07	7.93E-05	H2AFX, PCNA, S100A4, TK1, UBE2T
DEURIG_T_CELL_PROLYMPHOCYTIC_LEUKEMIA_UP	368	Genes up-regulated in T-PLL cells (T-cell prolymphocyti	5	0.0136	4.50E-07	1.01E-04	DKK1, H2AFX, HSP90AB1, SFN, THBS1
WILCOX_RESPONSE_TO_PROGESTERONE_UP	152	Genes up-regulated in primary cultures of ovarian surfa	4	0.0263	5.35E-07	1.13E-04	HMGCS1, INSIG1, MSMO1, TK1
HOFFMANN_LARGE_TO_SMALL_PRE_BII_LYMPHOCYTE_UP	163	Genes up-regulated during differentiation from large p	4	0.0245	7.07E-07	1.41E-04	DUT, H2AFX, TK1, UBE2T
PUJANA_BRCA2_PCC_NETWORK	423	Genes constituting the BRCA2-PCC network of transcrip	5	0.0118	8.93E-07	1.68E-04	DUT, H2AFX, HMGCS1, INSIG1, PCNA
ROME_INSULIN_TARGETS_IN_MUSCLE_UP	442	Genes up-regulated by 3 h of euglycemic hyperinsuline	5	0.0113	1.11E-06	1.96E-04	LAPTM4A, LDHB, MYL12B, UBB, XRCC6

Table S7. qRT-PCR primer sequences

Symbol	Name	Accession number	Forward	Reverse
ACTB	Actin, beta	NM_001101	5'-TTGCCGACAGGATGCAGAA-3'	5'-GCCGATCCACACGGAGTACT-3'
ADAM12	ADAM metallopeptidase domain 12	NM_003474	5'-AGCCACACCAGGATAGAGAC-3'	5'-CGCCTTGAGTGACACTACAG-3'
ALDH1A1	Aldehyde dehydrogenase 1 family, member A1	NM_000689	5'-TGTTAGCTGATGCCGACTTG-3'	5'-TTCTTAGCCCGCTCAACACT-3'
CD44	CD44 molecule	NM_000610	5'-AAGGTGGAGCAAACACAACC-3'	5'-AGCTTTTTCTTCTGCCCACA-3'
IL8	Interleukin 8	NM_000584	5'-TAGCAAAATTGAGGCCAAGG-3'	5'-AGCAGACTAGGGTTGCCAGA-3'
SERPINE1	Serpin family E member 1	NM_000602	5'-ATTGATGACAAGGGCATGGC-3'	5'-TCTGATTTGTGGAAGAGGCG-3'
VCAM1	Vascular cell adhesion molecule 1	NM_001078	5'-GTTGAAGGATGCGGGAGTAT-3'	5'-TTCATGTTGGCTTTTCTTGC-3'
