# Supplementary figures and images for: Metalloprotease-disintegrin ADAM12 actively promotes the stem cell-like phenotype in claudin-low breast cancer
Source: Mol Cancer. 2017 Feb 1;16:32. doi: 10.1186/s12943-017-0599-6 (PMC5288940; doi:10.1186/s12943-017-0599-6)

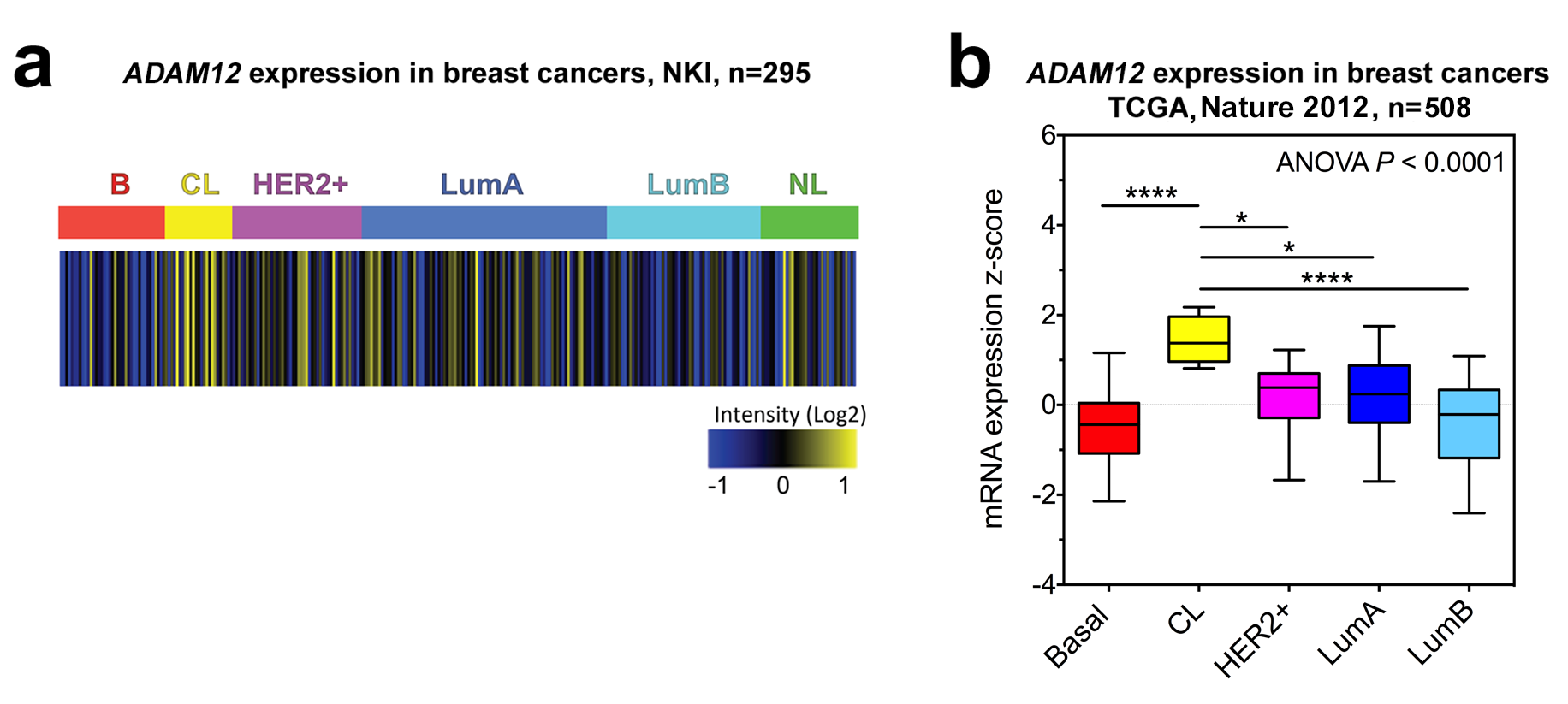

Supplement: Additional file 2: Figure S1. — ADAM12 mRNA is upregulated in claudin-low breast cancers. a Heatmap showing ADAM12 mRNA expression levels in 295 breast cancer patients from the Netherlands Cancer Institute (NKI) database [31]. Molecular subtypes of tumors are: B, basal; CL, claudin-low; HER2+, HER2-enriched; LumA, luminal A; LumB, luminal B; NL, normal-like. b Expression levels of ADAM12 in 508 breast cancer patients from The Cancer Genome Atlas (TCGA, Nature 2012 dataset) [33]. (TIF 4437 kb) [file 12943_2017_599_MOESM2_ESM.tif]

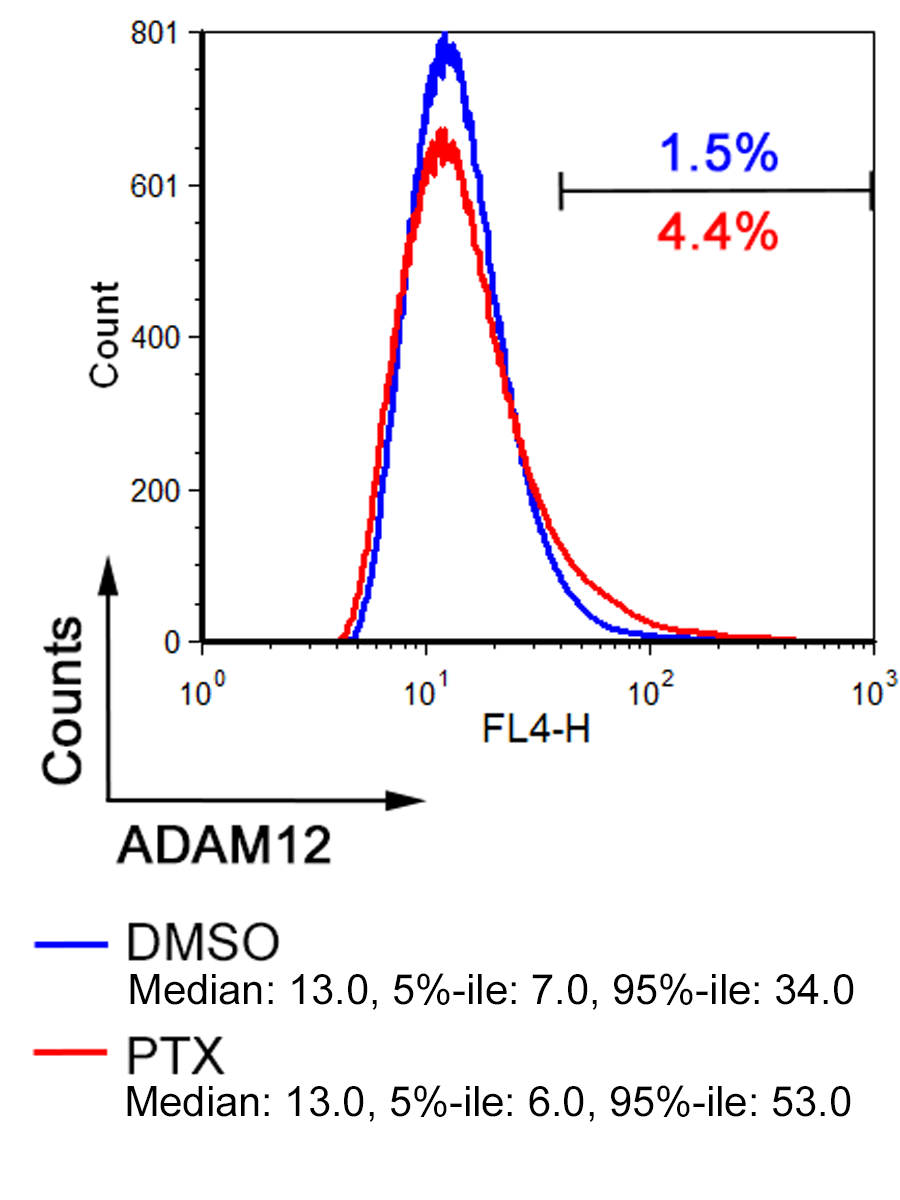

Supplement: Additional file 3: Figure S2. — Histogram analysis of a representative flow cytometry data showing the effect of 10 nM paclitaxel on ADAM12 expression in SUM159PT cells. Cells were treated for 6 days with DMSO (control) or with 10 nM paclitaxel (PTX), and then allowed to recover for 6 days without PTX. Live cells were stained with anti-ADAM12 antibody and propidium iodide (PI); only viable (PI-negative) cells were included in the analysis. To further identify ADAM12-positive cells, histogram subtraction of partially overlapping sample (anti-ADAM12 Ab) and control (isotype control Ab) histograms was performed using the FCS Express 4 software. Median fluorescence intensity of the resulting curves and the 5th and 95th percentiles for DMSO- and PTX-treated cells are shown. (TIF 3184 kb) [file 12943_2017_599_MOESM3_ESM.tif]

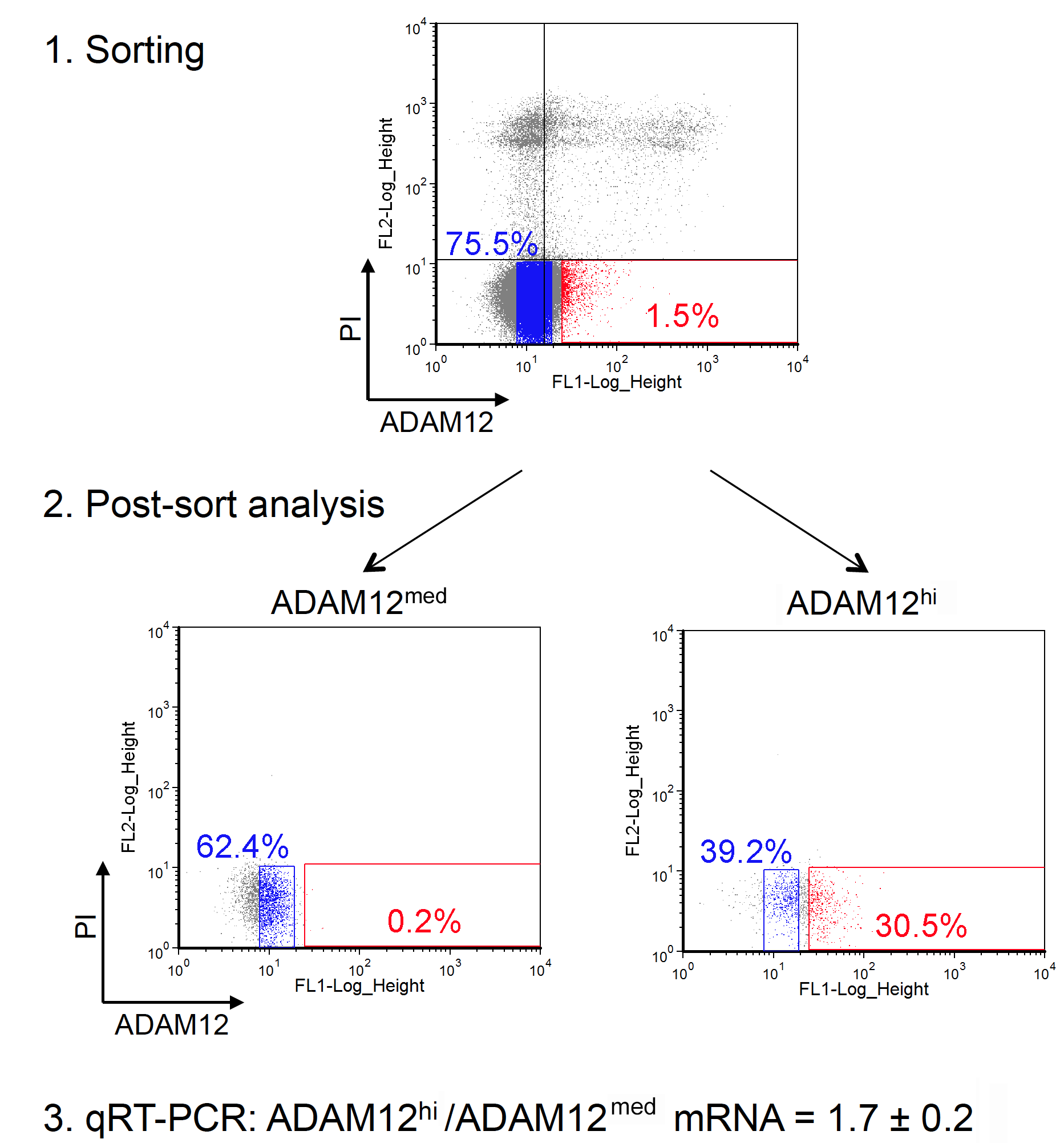

Supplement: Additional file 4: Figure S3. — Sorting ADAM12hi and ADAM12med subpopulations of SUM159PT cells. Live cells were stained with anti-ADAM12 antibody and propidium iodide (PI). A population of ~1.5% viable (PI-negative) cells with the highest expression of ADAM12, ADAM12hi, and a population with medium expression levels of ADAM12, ADAM12med, corresponding to the middle ~75% of all viable cells, were sorted. A subsequent post-sort flow cytometry and qRT-PCR analyses confirmed an enrichment of ADAM12hi and ADAM12med cells within sorted populations. (TIF 221 kb) [file 12943_2017_599_MOESM4_ESM.tif]

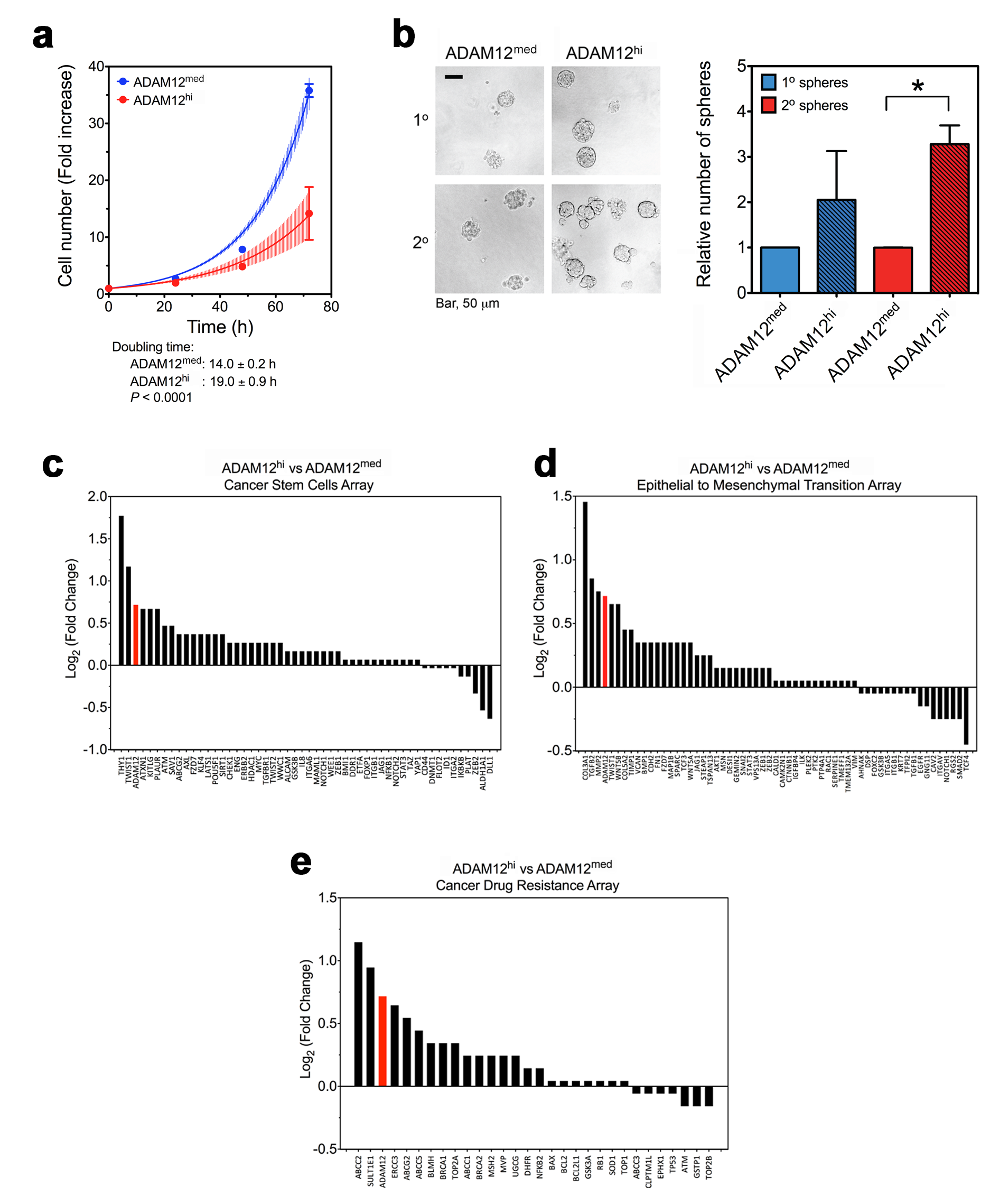

Supplement: Additional file 5: Figure S4. — ADAM12hi subpopulation of SUM159PT cells is enriched for cancer stem cell-like features. a Growth rates of sorted ADAM12hi and ADAM12med populations of SUM159PT cells. Sorted cells were plated in 96-well plates in triplicates and the relative numbers of live cells were determined 24 h -72 h later using the CellTiter-Glo viability assay. Graph represents mean values ± SEM from 2 independent experiments, as well as fitted exponential growth curves with 95% confidence interval. b Mammosphere formation by ADAM12hi and ADAM12med populations. Sorted cells were plated in ultra-low attachment plates in mammosphere medium containing 1% methylcellulose. After 10 days, primary (1o) mammospheres were collected, trypsinized, and single cell suspensions were seeded for secondary (2o) mammosphere formation. Mammospheres were visualized by phase contrast imaging, and the numbers of spheres with diameters > 50 μm were counted using ImageJ. The results are shown as means ± SEM obtained from 3 independent experiments. c-e, Relative expression of cancer stem cell markers (c), epithelial-to-mesenchymal transition markers (d), and cancer drug resistance genes (e) in ADAM12hi versus ADAM12med subpopulations of SUM159PT cells. Sorted cell populations were analyzed by qRT-profiling using RT2 Profiler PCR Arrays (Qiagen). Genes included in the arrays are shown in black, the data for ADAM12 is shown in red. (TIF 1278 kb) [file 12943_2017_599_MOESM5_ESM.tif]

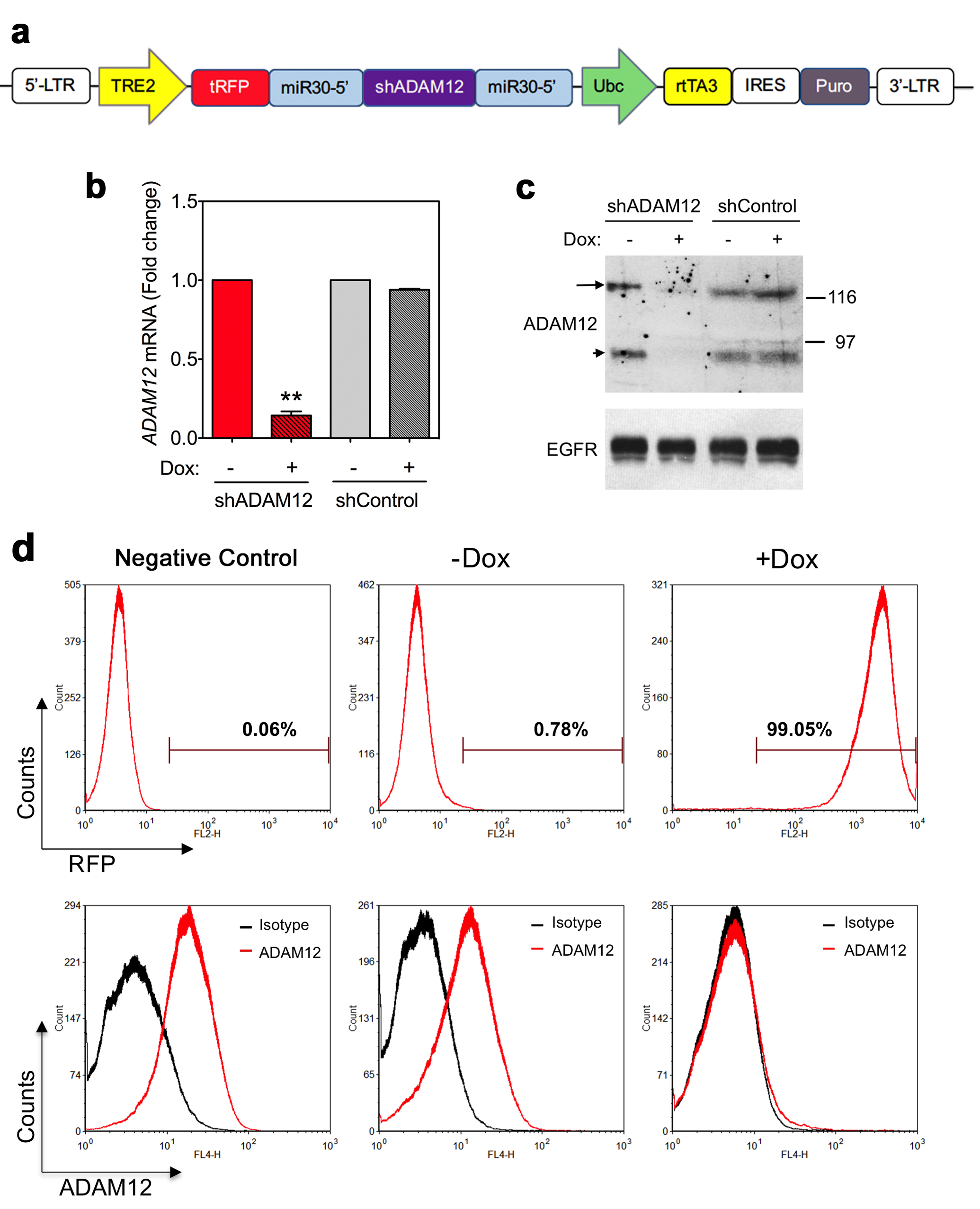

Supplement: Additional file 6: Figure S5. — Doxycycline-inducible ADAM12 knockdown in SUM159PT cells. a Diagram of the doxycycline-inducible lentiviral shRNA construct targeting ADAM12. 5’-LTR, 5’-long terminal repeat; TRE, tetracycline-inducible promoter; tRFP, turbo red fluorescent protein; miR30-5’ and shADAM12, micro-RNA-30-adapted shRNA targeting ADAM12; Ubc, human ubiquitin C promoter; rtTA3, reverse tetracycline-transactivator 3; IRES, internal ribosomal entry site; Puro, puromycin resistance gene; 3’-LTR, 3’-long terminal repeat. b, c, d Validation of ADAM12 knockdown. Stably transduced SUM159PT_shADAM12 and SUM159PT_shControl cells were incubated for 4 days with or without 1 μg/ml of doxycycline. b ADAM12 mRNA levels were quantified by qRT-PCR. c ADAM12 protein levels were determined by Western blotting after glycoprotein enrichment on concanavalin A agarose. EGFR serves as a loading control for glycoprotein-enriched fractions. Arrow, the nascent full length ADAM12; arrowhead, the processed form lacking the N-terminal pro-domain. d Cell surface expression of ADAM12 was examined by flow cytometry. (TIF 1149 kb) [file 12943_2017_599_MOESM6_ESM.tif]

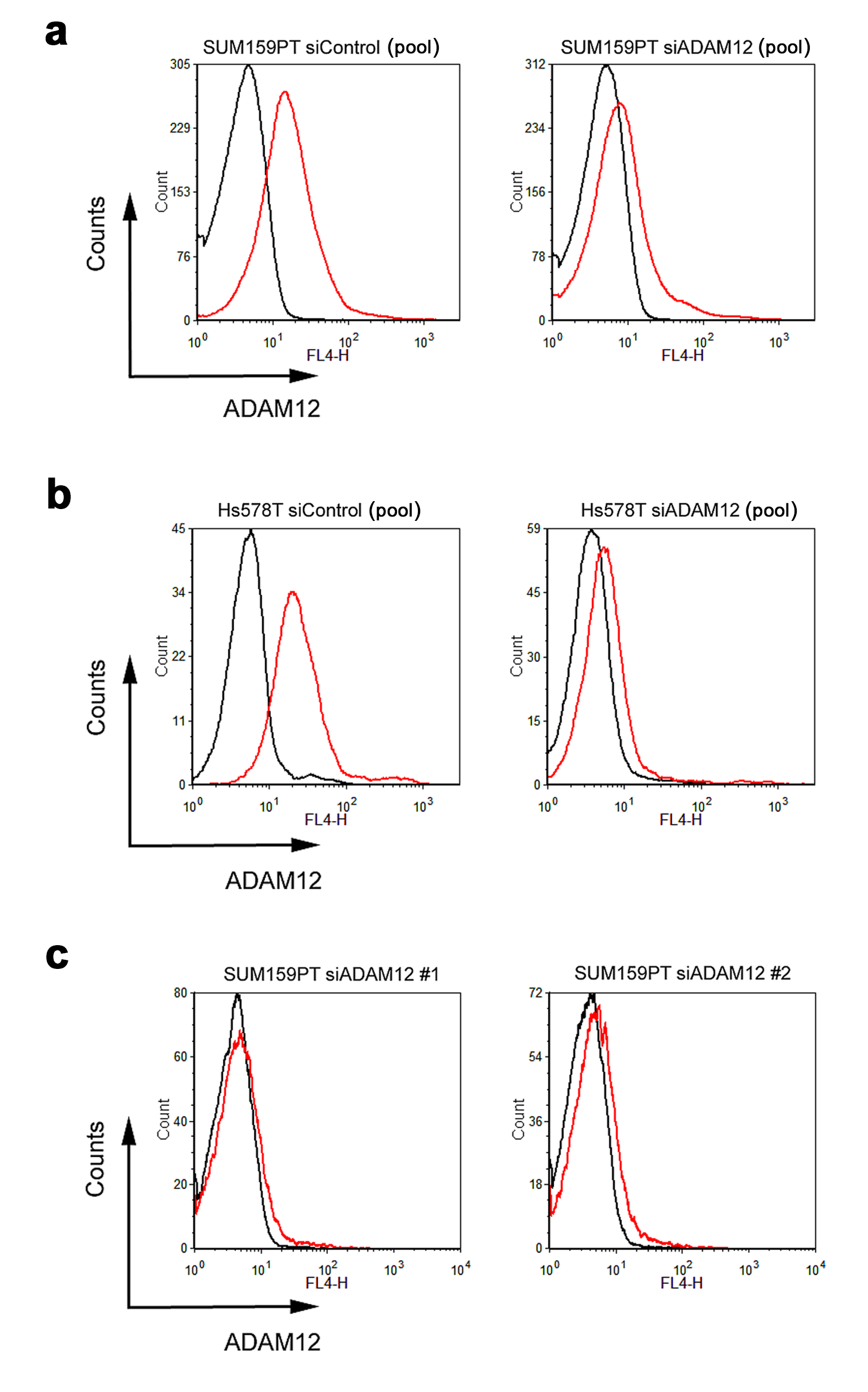

Supplement: Additional file 7: Figure S6. — ADAM12 knockdown using siRNAs. a SUM159PT cells were transfected with a pool of four siRNAs targeting ADAM12 or a pool of control siRNAs. b Hs578T cells were transfected with a pool of four siRNAs targeting ADAM12 or with a pool of control siRNAs. c SUM159PT cells were transfected with individual siRNAs, as indicated. Cell surface expression of ADAM12 was evaluated by flow cytometry. (TIF 556 kb) [file 12943_2017_599_MOESM7_ESM.tif]

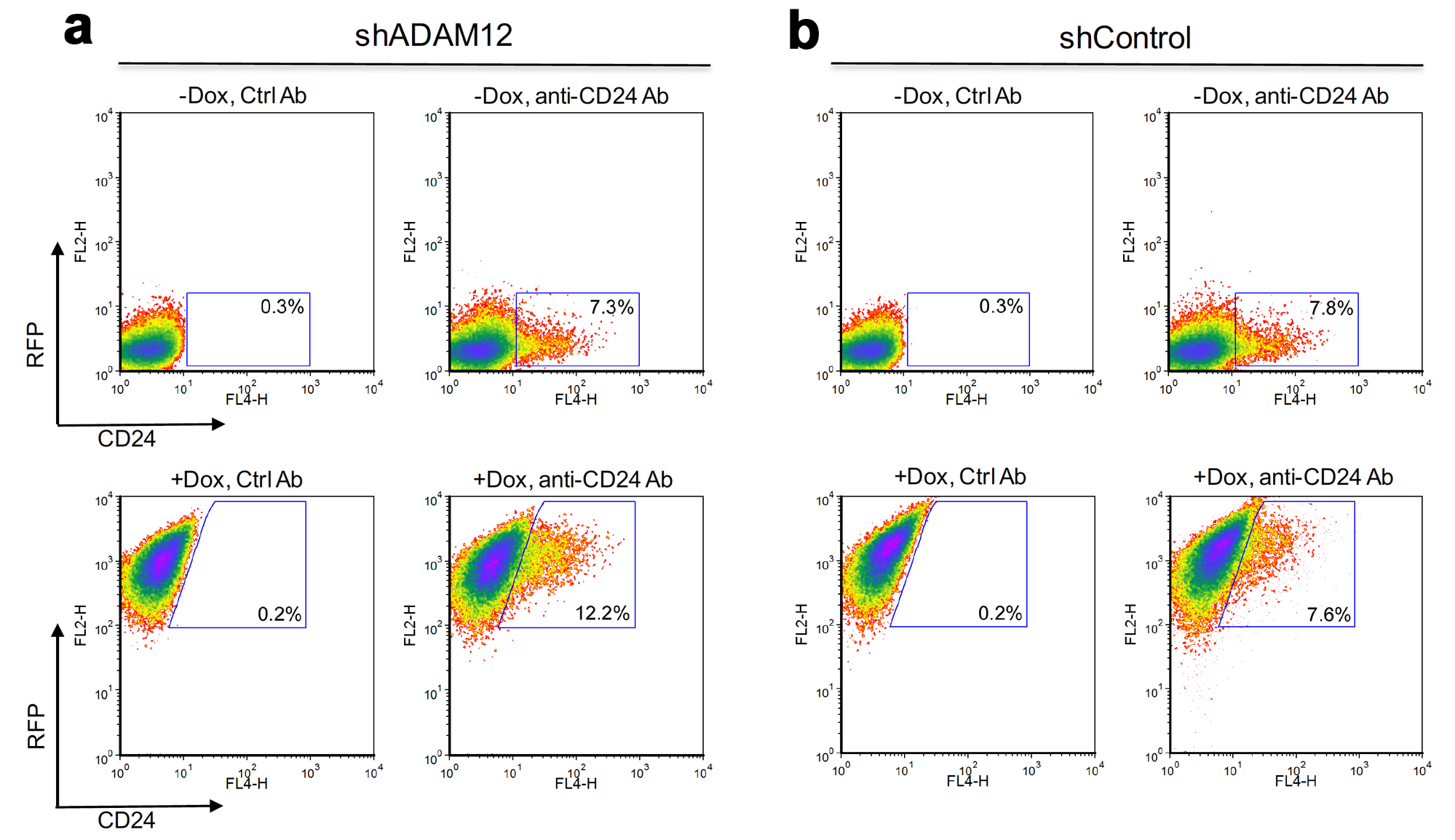

Supplement: Additional file 8: Figure S7. — Inducible ADAM12 knockdown in SUM159PT cells increases cell surface expression of CD24. SUM159PT_shADAM12 (a) or SUM159_shControl cells (b) were incubated for 4 days with or without 1 μg/ml of doxycycline. Cell surface expression was evaluated by flow cytometry. Percentages of CD24+ cells stained with FITC-conjugated anti-CD24 antibody, but not with isotype control antibody, are indicated. (TIF 706 kb) [file 12943_2017_599_MOESM8_ESM.tif]

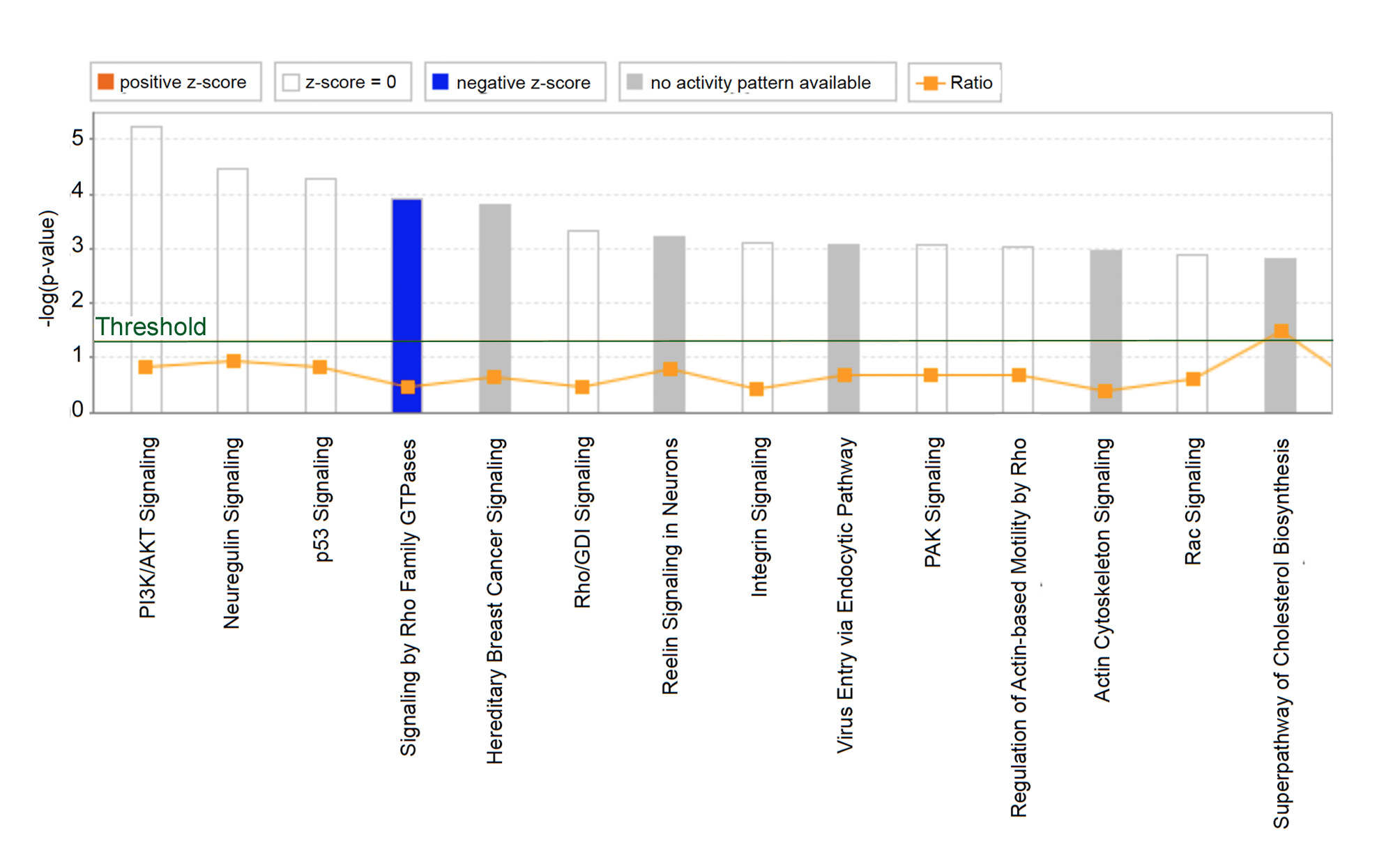

Supplement: Additional file 9: Figure S8. — Canonical pathways identified by IPA based on differentially expressed genes in response to ADAM12 knockdown. P-values for the canonical pathways are calculated by Fisher's exact test, right-tailed. The orange and blue colored bars indicate predicted pathway activation, or predicted inhibition, respectively (z-score). (TIF 572 kb) [file 12943_2017_599_MOESM9_ESM.tif]
